# Supplementary material for: Correction: Enhanced wave overtopping simulation at vertical breakwaters using machine learning algorithms
Source: PLoS One. 2025 Feb 3;20(2):e0318948. doi: 10.1371/journal.pone.0318948 (PMC11790087; doi:10.1371/journal.pone.0318948)
Supplement: S1 File — (PDF) [file pone.0318948.s001.pdf]

PLOS ONE

RESEARCH ARTICLE

Enhanced wave overtopping simulation at vertical breakwaters using machine learning algorithms

M. A. Habib[1], J. J. O'Sullivan[1,2], S. Abolfathi[3], M. Salauddin[1,2]*

1 UCD Dooge Centre for Water Resources Research, School of Civil Engineering, University College Dublin, Dublin, Ireland, **2** UCD Earth Institute, University College Dublin, Dublin, Ireland, **3** School of Engineering, University of Warwick, Coventry, United Kingdom

* md.salauddin@ucd.ie

Check for updates

OPEN ACCESS

Citation: Habib MA, O'Sullivan JJ, Abolfathi S, Salauddin M (2023) Enhanced wave overtopping simulation at vertical breakwaters using machine learning algorithms. PLoS ONE 18(8): e0289318. https://doi.org/10.1371/journal.pone.0289318

Editor: Zaher Mundher Yaseen, KFUPM: King Fahd University of Petroleum & Minerals, SAUDI ARABIA

Received: April 29, 2023

Accepted: July 16, 2023

Published: August 16, 2023

Copyright: © 2023 Habib et al. This is an open access article distributed under the terms of the Creative Commons Attribution License, which permits unrestricted use, distribution, and reproduction in any medium, provided the original author and source are credited.

Data Availability Statement: The raw data underlying the results presented in the study are available from www.overtopping.com. The datasets are freely available and authors did not have any special access privileges.

Funding: The author(s) received no specific funding for this work.

Competing interests: The authors have declared that no competing interests exist.

Abstract

Accurate prediction of wave overtopping at sea defences remains central to the protection of lives, livelihoods, and infrastructural assets in coastal zones. In addressing the increased risks of rising sea levels and more frequent storm surges, robust assessment and prediction methods for overtopping prediction are increasingly important. Methods for predicting overtopping have typically relied on empirical relations based on physical modelling and numerical simulation data. In recent years, with advances in computational efficiency, data-driven techniques including advanced Machine Learning (ML) methods have become more readily applicable. However, the methodological appropriateness and performance evaluation of ML techniques for predicting wave overtopping at vertical seawalls has not been extensively studied. This study examines the predictive performance of four ML techniques, namely Random Forest (RF), Gradient Boosted Decision Trees (GBDT), Support Vector Machines—Regression (SVR), and Artificial Neural Network (ANN) for overtopping discharge at vertical seawalls. The ML models are developed using data from the EurOtop (2018) database. Hyperparameter tuning is performed to curtail algorithms to the intrinsic features of the dataset. Feature Transformation and advanced Feature Selection methods are adopted to reduce data redundancy and overfitting. Comprehensive statistical analysis shows superior performance of the RF method, followed in turn by the GBDT, SVR, and ANN models, respectively. In addition to this, Decision Tree (DT) based methods such as GBDT and RF are shown to be more computationally efficient than SVR and ANN, with GBDT performing simulations more rapidly that other methods. This study shows that ML approaches can be adopted as a reliable and computationally effective method for evaluating wave overtopping at vertical seawalls across a wide range of hydrodynamic and structural conditions.

1. Introduction

The primary objective of coastal defences is to protect hinterland infrastructures and people from extreme sea levels, which typically arise from a combination of waves, high astronomical tides, and storm surges. Wave overtopping is observed at the crest of the coastal defence

Abbreviations: a) Parameters used in Fig 1 along with their units: A_c, armour freeboard relative to SWL (m); B, berm width (m); B_t, width of the toe (meter); cot (αd), angle of down slope (degree); cot (αu), angle of upper slope (degree); G_c, armour width (m); h_b, water depth on the berm (m);; H_{m0}, significant wave height at the toe of the structure (meter); h_t, water depth at toe of the structure (meter); q, Overtopping Discharge (m3/s per metre width); R_c, crest freeboard relative to SWL (meter); tan (αF), foreshore slope; tan (αf), foreshore slope; $T_{m-1,0}$, spectral wave period at toe of the structure (second); $\gamma_{t,d}$, roughness coefficient of the down slope; $\gamma_{t,u}$, roughness coefficient of the upper slope; **b) Parameters used for Prediction tasks with their units** B_t, Width of toe of structure (m); CF, Complexity-Factor of structure section; $cot\alpha_d$, cot of angle between structure slope downward berm and horizontal (-); $cot\alpha_{excl}$, cot of mean slope of structure calculated without contribution of berm (-); $cot\alpha_u$, cot of angle between structure slope upward berm and horizontal (-); G_c, Width of promenade (m); h, Water depth at toe of structure (m); h_b, Water depth on berm (negative means berm is above SWL) (m); $H_{m0\ d}$, Significant wave height at deep water (m); $H_{m0\ toe}$ / $H_{m0\ t}$, Significant wave height at toe of structure; h_t, Water depth at the toe of structure (m); mmm, Slope of foreshore; P_{ow}, Probability of overtopping per wave; R_c, Crest freeboard of structure (m); RF, Reliability-Factor of test; $T_{m\ d}$, Average wave period at deep water (s); $T_{m\ toe}$, Avg. wave period determined at toe (s); $T_{m-1,0\ d}$, Spectral wave period defined by m_{-1}/m_0 (s); $T_{m-1,0t}$, Spectral wave period at toe (s); β, Angle of wave attack (°).

structure when water exceeds the defence crest level and inundates the coastal lands on the lee-side of the structure (Fig 1). Wave-structure interactions are important in determining the resilience of coastal defences and their effectiveness in mitigating wave overtopping. As such, costal defences are designed based on an allowable limit for overtopping discharge. The assessment and prediction of wave overtopping discharge under different structural and hydrodynamic conditions has therefore been the focus of much research in recent years and continues to remain a critical design parameter, given the increased frequency of overtopping events that are predicted under current climate change trends [1, 2].

Multiple approaches lend themselves to determining the magnitude of wave overtopping discharge. For different defence structure geometry, physical, empirical and numerical methods have been applied to determine wave overtopping in terms of (a) overtopping discharge; (b) averaged overtopping discharge in a specific time per linear meter of the structure width; and (c) overtopping volume. Physical models have been developed to observe overtopping for a specific defence type (e.g., plain vertical walls, sloped walls, dykes, etc.) under defined wave conditions. Physically modelled data is generally used in further analysis and can underpin a sensitivity analysis to determine the influential overtopping parameters (see for example, [4–7]). With the advancement of computational fluid dynamics techniques, numerical models have been adopted to investigate wave overtopping and wave impact loads at sea defences (see for example, [8–11] presented a tool for predicting the magnitude of infragravity waves, which are long-period ocean waves in coastal areas with shallow foreshores and dikes. The tool is based on numerical models and considers factors such as wave height, water depth, and foreshore slope to make predictions. Afterwards, [12] discussed the refinement of a mathematical formula for modelling wave overtopping and runup at coastal structures, such as breakwaters and sea walls. Empirical formulae are developed based on physical and numerical datasets to provide predictive relations for estimating wave overtopping for a range of structural geometries and hydrodynamic conditions (see for example, [13–15] and [3]]).

The EurOtop [3] overtopping manual is a comprehensive and state-of-the-art design manual for a range of coastal structures including seawalls, breakwaters, and revetments for various geometries. The manual encompasses design guidance to estimate potential overtopping at coastal structures exposed to a comprehensive range of wave actions. The open source data used in the manual includes laboratory experiments and field observations of wave actions and their impacts on coastal structures, and amounts to *c*. 18,000 tests. EurOtop [3] was developed by a team of coastal engineering experts from various European countries. The latest version

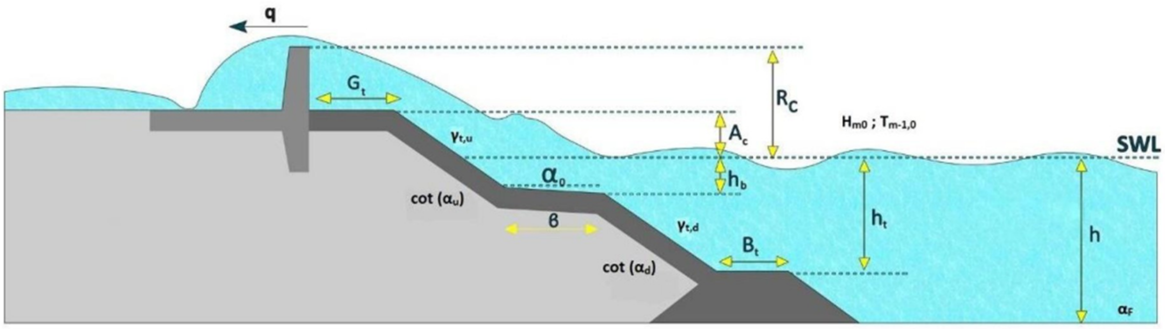

Fig 1. Schematic of parameters used in describing wave overtopping discharge at sea defences (adopted from [3]). See glossary for further description of the parameters.

https://doi.org/10.1371/journal.pone.0289318.g001

of the manual (and dataset) was published in 2018, and it remains a primary guidance for coastal engineers across the world to design resilient coastal structures.

The combination of advances in computational power, availability of high-resolution datasets, and efficiency of computational algorithms, has added momentum to the application of data driven approaches for predicting wave overtopping characteristics. In this regard, the use of Machine Learning (ML) algorithms, where the algorithm learns complex non-linear patterns from datasets to map input features to an output quantity, have gained interest in the coastal engineering community. MLs have shown superior performance in addressing a range of complex water and environmental problems compared to traditional statistical approaches of classification and regression analysis (e.g., [16–24]). Artificial Neural Networks (ANN) have been used for estimating wave overtopping for a range of defences [4, 25, 26] and have been endorsed in [3]. More recently, [27, 28] adopted Gradient Boosted Decision Trees (GBDT), a variant of a decision tree model, to study overtopping using the data from the CLASH [29] database and other physically based experimental data. The results from the GBDT model were shown to compare favourably to corresponding predictions from ANN models and empirical formulations. Other applications of decision trees for overtopping predictions are investigated by [30–32]. Random Forest (RF) and Support Vector Machines Regression (SVR) models are also shown to outperform ANNs for predictions of wave overtopping [33, 34]. ML-based predictive modelling studies for wave overtopping highlight the necessity of a robust methodological approach for choosing appropriate ML techniques to achieve high levels of performance in overtopping prediction [35].

Here we present a comprehensive assessment of four widely adopted ML algorithms in prediction of wave overtopping from vertical breakwaters. Random Forest (RF) and Gradient Boosted Decision Tree (DT) that are derivatives of general decision tree models, together with Support Vector Machine Regressor (SVR) and Artificial Neural Network (ANN) are implemented in this study to predict mean overtopping rates at vertical defences. The EurOtop (dataset [3] comprising extensive overtopping data from physical modelling tests for vertical sea defences is utilised to develop, train, and test the ML algorithms. The dataset was split to ensure an unbiased evaluation of model performance. Each ML model is first trained and validated on 70% of the dataset (EurOtop data on vertical wall) and subsequently tested on the remaining 30%, which remained unseen for the ML algorithm during the training and validation phase. The predicted overtopping rates are compared to the measured values and detailed statistical analysis is performed using five statistical error metrics. The computational time was also compared. The paper examines the robustness of the four ML-based algorithms for their appropriateness in for modelling wave overtopping at coastal defences, and for the first time, investigates the applications of optimization techniques such as hyperparameter tuning and feature selection methods to enhance both statistical metrics and physical consistency. A methodological framework is developed and adopted for a meaningful comparison between the predictions of the ML algorithms. It is shown that use of advanced feature selection methods implemented in this study can replace the existing time-consuming permutation method of finding the best set of features for the overtopping prediction tasks in the datasets.

2. Materials and methods

2.1 Decision trees

Decision Trees (DTs) are supervised algorithms widely adopted for prediction tasks in both classification and regression problems. A typical structure of a DT is shown in Fig 2, where the black and white circles represent two distinct set of data features. The entire sample containing the features are inputted into the root node. The features are then separated in the interior

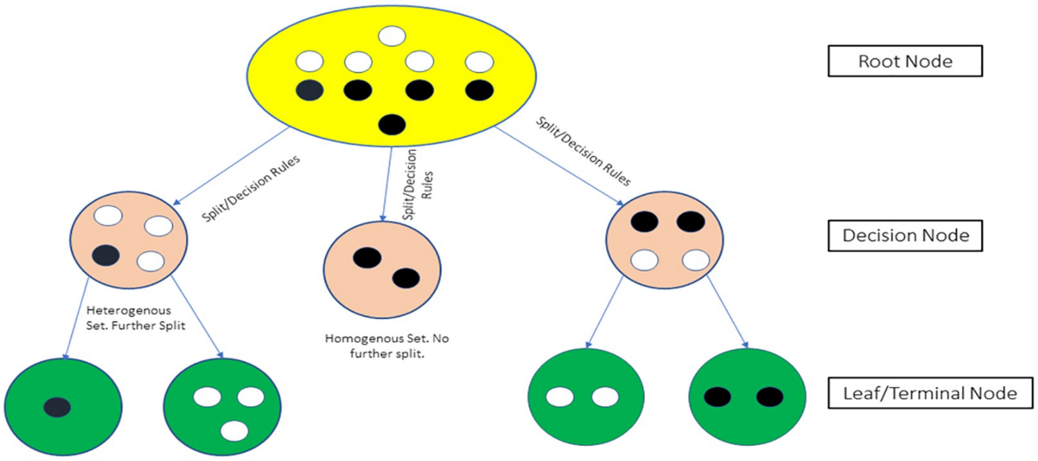

Fig 2. Typical structure of a decision tree (DT).

https://doi.org/10.1371/journal.pone.0289318.g002

nodes according to decision-rules that the algorithm develops based on the training on the dataset. The leaf nodes in DTs produce the outputs or the predicted quantities (for regression trees). The final output of a DT is achieved through multiple iterations of the algorithm and is expressed as an average of all the outputs from successive iterations. The reduced complexity of DT models and the fact that their performance is largely independent on the 'noise' and the non-linearity of input data, together with the ability of these models to handle both categorical and numerical data has contributed to their wide popularity [36, 37].

If we consider a set of training features (such as the overtopping parameters) $x_i \in R^n$; $i = 1$ *to* l and label vectors (such as q, the overtopping discharge) $y \in R^l$, the function of a decision tree is to repeatedly split the space of the training vectors so that they have the same labels. The split points are called 'nodes'. Suppose Q_m is a node with n_m features, splitting criteria $\theta = (j, t_m)$ where j is a feature set, and t_m is the threshold, then following the split, two subsets are formed which are $Q_m^{left}(\theta)$ and $Q_m^{right}(\theta)$. The quality of each split is computed using the impurity or the loss function $H()$ which is different for classification and regression tasks. The minimum of the function $G(Q_m,\theta)$ (Eq 1) is θ^* (Eq 2) to be reached with successive splits. (Pedregosa et al., 2012).

$$G(Q_m, \theta) = \frac{n_m^{left}}{n_m} H\left(Q_m^{left}(\theta)\right) + \frac{n_m^{right}}{n_m} H\left(Q_m^{right}(\theta)\right) \quad (1)$$

$$\theta^* = argmin\, G(Q_m, \theta) \quad (2)$$

The split continues until the minimum of the loss function is achieved or until $n_m < n_{min}$ or $n_m = 1$.

For regression tasks, such as the prediction of overtopping discharge from overtopping parameters (see Fig 1), the split at the nodes is based on the Mean Square Error (MSE) or *L2* error, the Half Poisson (HP) deviance and the Mean Absolute Error (MAE) of the predicted and actual values (i.e. labels). In this study the MSE approach is applied given that the MSE computation is faster than the HP deviance. The loss function $H(Q_m)$ is then computed by Eq 3:

$$H(Q_m) = \frac{1}{n_m} \sum_{y \in Q_m} (y - \bar{y}_m)^2 \quad (3)$$

where, $\bar{y}_m$ is the mean of the predicted values and is set according to Eq 4:

$$\bar{y}_m = \frac{1}{n_m} \sum\nolimits_{y \in Q_m} y \tag{4}$$

Poor performance of individual DTs can be associated with overfitting problem, an issue that is typically addressed by bagging and boosting approaches. To address the overfitting problem, two derivatives of DTs; Random Forests and Gradient Boosted Decision Trees (GBDT) are proposed.

Bagging or bootstrap aggregation generates ensemble DT regressors that account for all predictions from individual DTs and give predictions of a quantity based on the majority voting of the ensembles. An example of a bagging based-DT algorithm is Random Forest (RF). In a RF, an ensemble of DTs is constructed using a subset of all features which are randomly selected. Following this, predictions from each DT in the ensemble are computed and a final predicted quantity is based on the best predictor model. The steps involved in a RF model can be summarized as follows (Fig 3):

Step 1: *Data Preparation and Splitting*

- Load the training data in an accessible format to the algorithm
- Tackle missing data through imputation methods and check for outliers

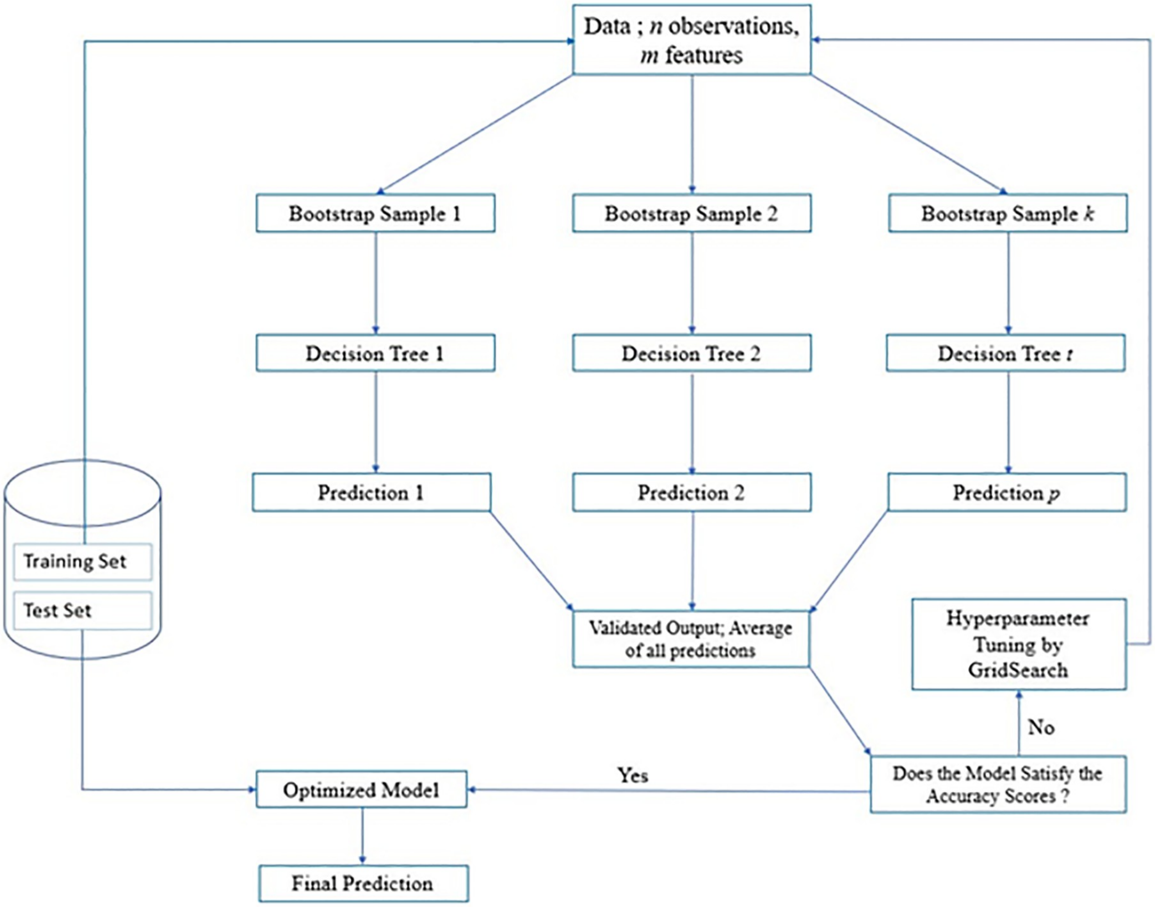

Fig 3. The structure and workflow of a RF algorithm.

https://doi.org/10.1371/journal.pone.0289318.g003

- Separate the predictor variables (input features) and the target variable (output feature)
- Perform split of training and test data (70% and 30% respectively)

Step 2: *Bootstrap Sampling*

- Generate subsets of the training data with replacement using a method called bootstrap sampling

Step 3: *Building Ensemble Decision Trees*

- Ensembles of DTs (Decision Trees 1, 2. . .t; Fig 3) are generated
- Each subset generated using bootstrap sampling is assigned to a regression based Decision Tree (DT).
- The DT splitting criteria is set using a random selection of predictor variables.
- The number of features to take into account for each split is typically equal to the square root of the total number of features, although this may be altered using hyperparameter tuning.

Step 4: *Training, Validation and Prediction on Training Set*

- The ensemble DTs are validated on the training data. Statistical parameters (such as mean and median) of the relevant leaf nodes in each decision tree are used to deduce the output value. The overall output value is the average of the individual trees in the ensemble.

Step 5: *Hyperparameter Tuning*

- Utilize assessment measures like mean squared error (MSE), mean absolute error (MAE), or coefficient of determination (R-squared) to rate the effectiveness of the random forest regression model.
- Adjust the random forest's hyperparameters such as the number of trees, maximum depth of individual trees or the amount of characteristics to take into account at each split and re-run the above steps.

Step 6: *Deployment to the test set*

- The optimized algorithm is then applied to the test set and the performance was evaluated using statistical metrics.

In boosting, prediction errors are minimised by differentiating the loss function (Eq 2) to reach the minimum value. During the training process and through multiple iterations, different weights and coefficients are associated with the values of the input variables to optimize the loss function to a minimum value. The adjustment of the loss function after each iteration is called the gradient descent. In optimising the loss function, the accuracy of predictions from the DT is improved in a process referred to as boosting, with the resulting DT model being the Gradient Boosted Decision Tree (GBDT) [38]. Fig 4 summarizes the following steps that constitute the GBDT algorithm.

Step 1 is similar to that of RF.

Step 2: *Building Decision Trees*

GBDT is characteristically a Decision Tree and hence the setting up of the trees are similar to what was discussed in Step 3 of the RF methodology. Hyperparameters like the learning rate, maximum tree depth, number of estimators, and regularization parameters are initialized in this step.

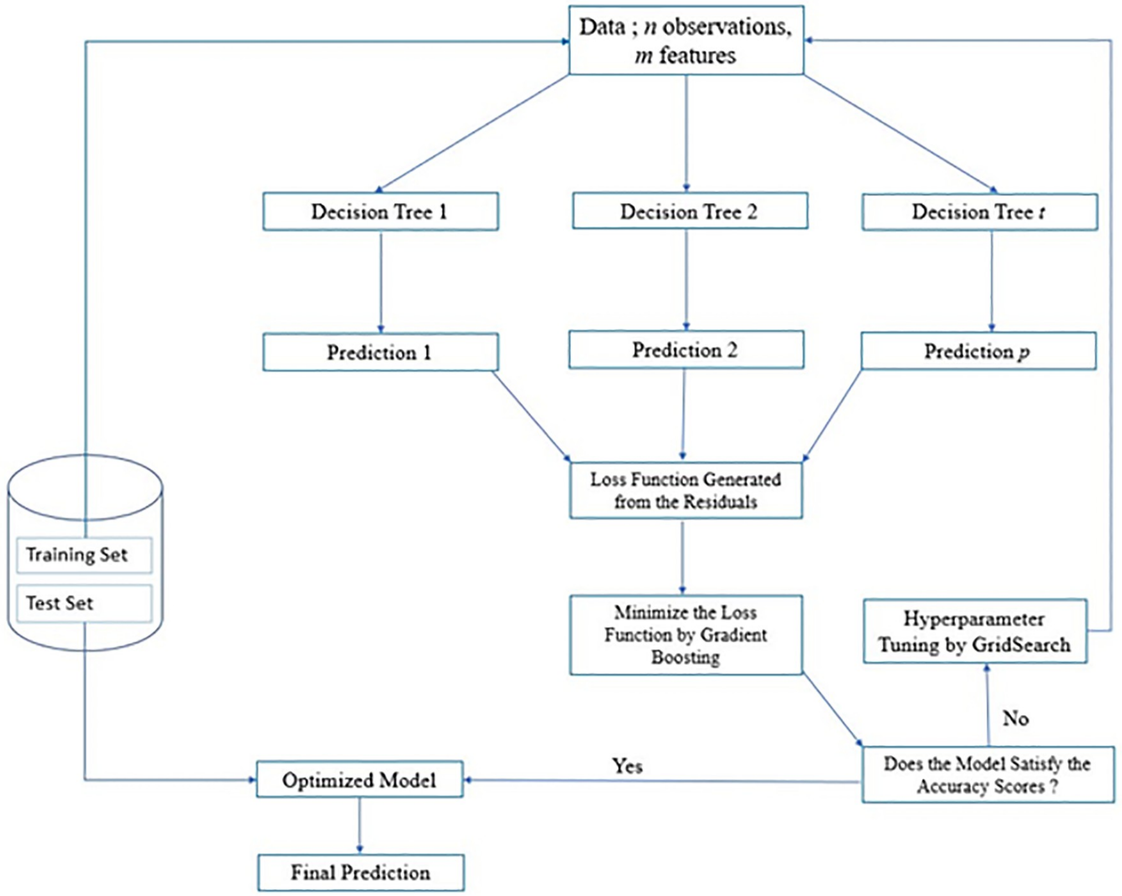

Fig 4. The structure and workflow of a GBDT algorithm.

https://doi.org/10.1371/journal.pone.0289318.g004

Step 3: *Optimizing the Loss Function*

Following an initial run, the GBDT model will create an ensemble of DTs and the discrepancies between the actual and predicted values are translated into a loss function. The model then works to minimize this loss function during the training step.

Step 4: *Training and Validation*

The model is trained and validated on the training set and performance is estimated using statistical metrics.

Step 5: *Hyperparameter Tuning*

In this step, the hyperparameters can be tuned by the user to best fit on the training data. Hyperparameter tuning is performed based on the performance evaluation on the training data.

Step 6: *Deployment to the test set*

Based on satisfactory results from the training and validation step, the model is then applied to the test set.

2.2 Support vector machines

Support Vector Machine (SVM) is another method of supervised ML that can be used for both classification and regression tasks. The main target of a SVM is to build a hyperplane in a *n*

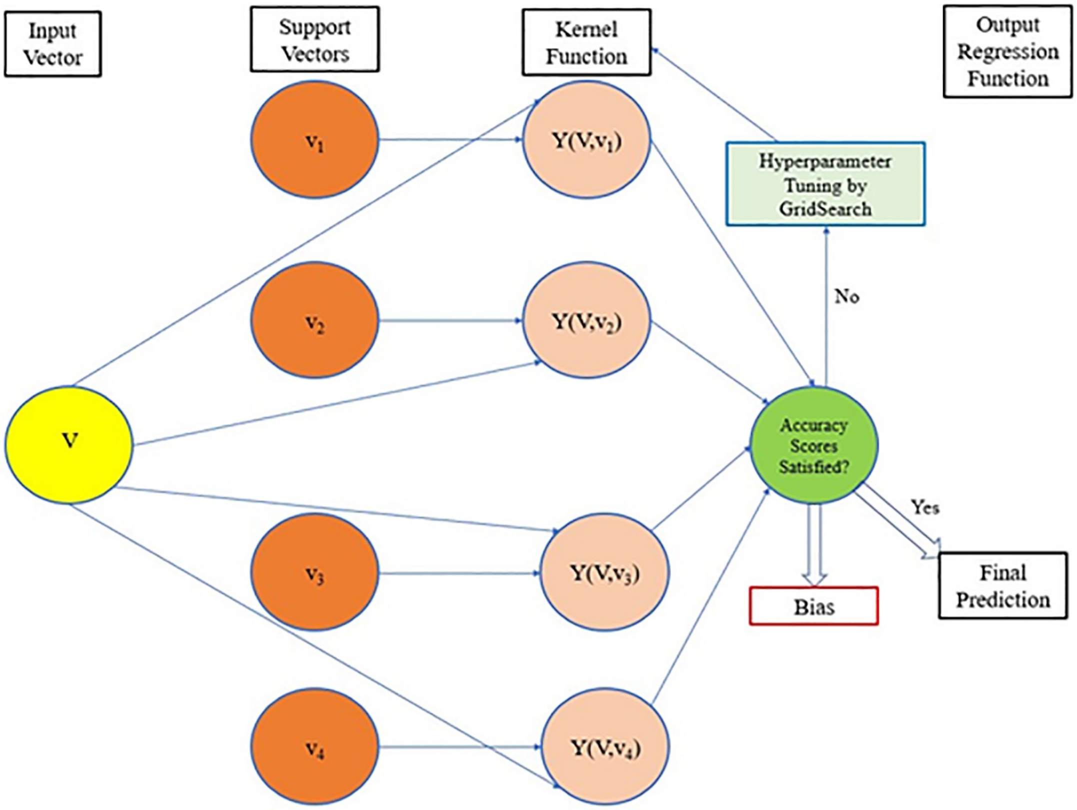

Fig 5. The structure of a SVR algorithm.

https://doi.org/10.1371/journal.pone.0289318.g005

dimensional space where each discrete point will be representing the feature data. The data points that fall close to either side of the hyperplane are called support vectors. The support vectors are then used to predict an output. SVMs were initially developed to predict categorical or discrete values. The typical structure of a SVR algorithm is illustrated in Fig 5. The algorithm can be summarized in the following steps.

Step 1 involves the usual process of assemble the dataset with the target variables and the input features. Pre-processing tasks like data cleansing, feature scaling, or addressing missing values are carried out as the SVR algorithm is susceptible to missing values. The dataset is then split into a training and test set and transformed to input vectors (See Fig 5).

Step 2: *Defining the Support Vectors*

The model parameters are defined which include selecting the kernel function. The regularization parameter (C) and kernel-specific parameters (such as degree for polynomial kernels and gamma for RBF kernels) are additional hyperparameters that are defined in this step.

Step 3: *Training and Validation*

In this step, the model's performance is evaluated for the initial set of hyperparameters.

Step 4: *Hyperparameter Tuning*

The hyperparameters are altered to tailor the algorithm by altering the parameters inside the kernel function.

Step 5: *Deployment to the test set*

The optimized SVR model is then applied to the test set.

A sub-class of SVMs, namely the Support Vector Machine Regressor (SVR) deals with predicting continuous values. SVR models include several features that make them an ideal choice for solving both linear and non-linear prediction problems [39, 40]. SVR models are considered to be more efficient in reducing errors during predictions [39, 40]. In linear SVR, unlike linear regression, the hyperplane takes the shape of a tube, known as an ε-tube and the regression line falls in the middle of this convex tube. The SVR function $y = f(x)$ in Eq 5 [41] is then optimised to have the flattest ε-tube that defines most of the training data. Any data point that falls in the ε-tube is disregarded in the error calculations, while only the data points that fall outside the tube are included, enabling tuning of the margin of error of the regression model. The regression error is then measured by calculating the distance between the data points and the ε-tube. The SVR is known for high generalisation ability over unseen data and can perform prediction tasks with significant accuracy regardless of the number of features in the dataset [41, 34].

$$y = f(x) = < w, x > +b = \sum\nolimits_{j=1}^{M} w_j x_j + b,\ y, b \in R,\ x,\ w\ \in\ R^M \tag{5}$$

where w is the support vectors, and 'M' is the degree of polynomial used in the approximation function. The loss function is calculated by Eq 6:

$$\min \frac{1}{2} ||w||^2 + C \sum_{i=1}^{N} \varepsilon_i + \varepsilon_1^* \tag{6}$$

where N is the number of slack variables, C is a regularization factor that can be tuned to alter the flatness of the hyperplane, and ε_i and ε_1^* are slack variables that measure the distance of the outliers from the ε-tube. In this study, a non-linear SVR is developed for dealing with non-linear relationships between the features and the target variable. In the case of non-linearity, the feature data is mapped onto a higher dimensional hyperplane known as kernel space, contributing to enhanced accuracy of the model.

The kernel space is defined by $k(x_i, x_j)$. This study adopts a Gaussian Radial Basis Function as described in Eq 7. The Gaussian RBF can accommodate an infinite number of feature dimensions as it follows a Taylor Series expansion and is widely accepted as an appropriate kernel for datasets where intrinsic characters of the parameters are unknown or difficult to trace (e.g. overtopping datasets) [42].

$$k\left(x_i,\ x_j\right) = exp\, exp\left(-\frac{||x_i,\ x_j||^2}{2\sigma^2}\right) \tag{7}$$

where σ is a kernel parameter.

2.3 Artificial neural networks

Artificial Neural Networks (ANNs) are inspired by the biological neurones and were first applied by [43]. ANNs make predictions by mapping inputs to outputs assigning weights to particular inputs and estimating a loss function. ANNs have been used for prediction of overtopping over a range of coastal defence structures described by the CLASH dataset [29] and [3, 26, 44, 45, 46]. [25] demonstrated the appropriateness of ANN to perform data-driven analysis on overtopping datasets with a large number of parameters, where the inter-relationship between the parameters is unknown or difficult to deduce. The layered structure of an ANN comprising input, hidden, and output layers is shown in Fig 6. The input layers initially receive data from the feature sets. The exchange of information only takes place between the neurones

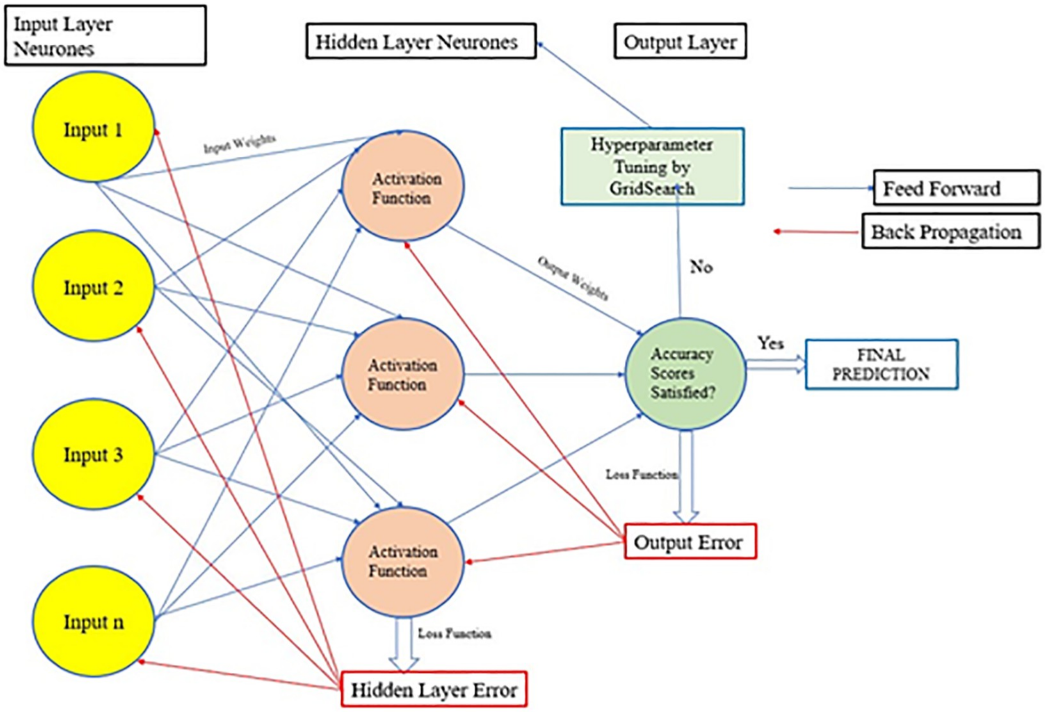

Fig 6. Structure of a feed forward back propagation ANN.

https://doi.org/10.1371/journal.pone.0289318.g006

and not within them. The hidden layers of the model (or 'black-box') perform the operation of applying activation functions and appropriate weights to the input values. The hidden layers compute the dependent feature(s) from the independent features in the input layers and pass these to the final, or output layer, of the network [47]. In the current study, a feed-forward and back propagation ANN algorithm is adopted for overtopping prediction. As such, the error rates were minimised by optimising the loss functions through a combination of feed-forward (passing of information from Input to Hidden to Output Layers) and back propagation (Output to Hidden and Hidden to Input Layers), until a certain threshold of allowable error rate is achieved. The error rate is an estimation of the extent of which the weights and biases are reconfigured during the back propagation step. This is carried out by assigning new weights and activation functions in the hidden layers. The number of hidden layers can be optimised according to the complexity of the input data in order to minimise errors in predictions [39, 40]. Two hidden layers, each consisting of 500 neurones were used to construct the ANN model in this study. Hyperparameter tuning was adopted to ensure that the best combination of parameters curtailed for the overtopping dataset are used for the overtopping prediction tasks.

2.4 Evaluation metrics

The performance of the ML algorithms in terms of predicting the overtopping quantity, described as discharge per unit width of the structure 'q', was evaluated by comparing the predictions to measured values and determining statistical metrics including Coefficient of Determination (R^2), Root Mean Square Error (RMSE), Root Mean Squared Logarithmic Error (RMSLE), Mean Absolute Error (MAE), and Relative Absolute Error (RAE).

The Coefficient of Determination (R^2 in Eq 8), for a set of model estimates, defines the proportion of the variation in the dependent variable that is predictable from the independent variables and as such, is a measure of the overall model performance [48]. R^2 values range from 0 to 1, with 1 representing the perfect predictive performance of a model.

$$R^2 = 1 - \frac{\sum (y_i - \hat{y}_i)^2}{\sum (y_{i-} - y)^2} \tag{8}$$

where y_i, $\hat{y}_i$ *and* $\bar{y}$ are observed values, predicted values, and mean of all observed values, respectively.

The Root Mean Square Error (RMSE) captures the standard deviations between the observed and predicted values (Eq 9):

$$RMSE = \sqrt{\frac{1}{N}\sum_{i=1}^{N} (q_A - q_P)^2} \tag{9}$$

where, q_A and q_P are the actual and predicted overtopping volumes, respectively.

The Root Mean Squared Logarithmic Error (RMSLE) in Eq 10 measures the differences between actual and predicted values expressed in logarithmic scale, and provides a fair treatment of both small and large errors where there may be outliers in the predicted values:

$$\text{RMSLE} = \sqrt{(log\, log\, (q_A + 1) \; - log\, log\, (q_P + 1))^2} \tag{10}$$

The Mean Absolute Error (MAE) is calculated by measuring the magnitude of the differences between the actual and predicted values and is averaged over the number of observations (Eq 11):

$$\text{MAE} = \frac{1}{N}\sum_{1}^{N} |q_A - q_P| \tag{11}$$

Finally, the Relative Absolute Error (RAE), ranging from 0 to 1 in order of decreasing accuracy, is obtained by dividing the total absolute error by the absolute difference (Eq 12):

$$\text{RAE} = \frac{\sum_{i=1}^{N} |q_A - q_P|}{\sum_{i=1}^{N} |q_A - q_{Av}|} \tag{12}$$

where $q_{Av} = \frac{1}{N}\sum_{i=1}^{N} q_A$.

2.5 hyperparameter tuning

Hyperparameter refers to any parameter of an ML algorithm that can be altered or tuned by the user, whereas model parameters (e.g., coefficients of mapping functions) cannot be accessed by the user. Hyperparameter tuning ensures that the ML algorithm is tailored for a particular input data and is an important feature for reducing risks of overfitting. Hyperparameter tuning was performed for all the ML models in this study following the scikit-learn library suggestions [36].

Table 1 shows the optimal hyperparameters for the SVR, RF, GBDT, and ANN models. The *C* term in Table 1 is a regularisation parameter of the SVR algorithm. The kernel is the engine of the algorithm and is a function that maps the input parameters to the output. The kernels considered in this study include linear, polynomial, and RBF. Gamma (in Table 1) is a coefficient of the kernel function. The optimal parameters were achieved through the combination of grid search and a *k*-fold Cross Validation (CV) [36]. A grid search is the term used for the

Table 1. List of the hyperparameters and their corresponding best values.

Algorithm	Hyperparameters and Typical Values	Best Values
SVR	C': 5, 10, 15; 'kernel': linear, rbf, poly; 'gamma': auto, scale	C': 15; 'kernel': rbf, 'gamma': auto
RF	n_estimators': 800, 1000, 1500<br>'min_samples_split': 2, 5, 10<br>'min_samples_leaf': 1, 2, 4<br>'max_features': auto, sqrt<br>'max_depth': 10 to 110<br>'bootstrap': True, False	n_estimators': 800,<br>'min_samples_split': 2,<br>'min_samples_leaf': 1,<br>'max_features': 'auto',<br>'max_depth': 100,<br>'bootstrap': True
GBDT	max_depth': 3, 5, 6, 10, 15, 20;<br>'learning_rate': 0.01, 0.1, 0.2, 0.3;<br>'subsample': 0.5, 1.0, 0.1;<br>'colsample_bytree': 0.4, 1.0, 0.1;<br>'colsample_bylevel': 0.4, 1.0, 0.1;<br>'n_estimators': 100, 500, 1000	max_depth': 6; 'learning rate': 0.1; 'subsample': 1.0; 'colsample_bytree': 0.7;<br>'colsample_bylevel': 1.0; 'n_estimators': 1000
ANN	activation': 'relu', 'sigmoid'; 'batch_size': 32, 64, 128; epochs': 10, 15, 20, 'alpha': 0.0001, 0.0005	activation': 'relu', 'batch_size': 64, 'epochs': 20, 'alpha': 0.0001

https://doi.org/10.1371/journal.pone.0289318.t001

collective number of hyperparameter combinations. A *k*-fold CV is the operation that splits a particular training set into subsets called 'folds'. The ML algorithms are first validated on these folds before being applied to the test set. This is to ensure the algorithm captures all the variance and patterns of the training set.

The hyperparameters of RF (Table 1) are mostly the functional units of a decision tree network. The 'n_estimators' is the number of trees that collectively form the RF. Features such as 'max_depth', 'min_samples_split' serve to reduce overfitting. Unlike the grid search with CV used in SVR model development, a random search with CV hyperparameter tuning method is adopted for the RF model, which yielded higher accuracy compared to grid search CV for the data used in this study. The Randomised Search (RS) CV uses a fixed number of random combinations of hyperparameters, unlike the Grid Search (GS) CV that employs all possible combinations.

The ideal hyperparameter configuration for GS is often identified by assessing the model's performance for each set of parameter values defined in the grid. This information can be accessed using the 'cv_results' attribute in scikit-learn's package, as k-fold cross-validation was also implemented. Each configuration's performance is measured using a performance metric, such as accuracy or mean squared error, and the configuration with the greatest (or lowest, depending on the metric) performance is selected eventually. The list of best values for the GS can be retrieved using the 'best_params_' function.

In the case of RS, for each random parameter configuration, the model is trained and assessed, and the optimal configuration is chosen based on the performance measure. The retrieval process of the best parameters are similar to what has been discussed about GS.

Functionally, RF and GBDT are both decision trees with the exception that the latter relies on gradient boosting as previously discussed. The hyperparameters of GBDT (Table 1) deal with the size of the decision tree that will be a best fit for the data used. The 'learning_rate' of a GBDT is an important criterion that reduces model overfitting by setting the weights of individual features in the boosting process to converge the error in the loss function. The 'max_depth' feature also contributes to reducing overfitting by limiting the number of nodes in the trees. Random search with five steps CV is shown to have the best performance for the GBDT model developed in this study.

ANN models have limited scope to work with the hyperparameters, as most of the model parameters, such as altering the learning rates, cannot be manually changed [49] In this study, the learning rate 'alpha' was altered using both GS and RS methods and the best model was

selected on the model loss criteria. The typical values for hyper tuning the ANN were based on existing literature (see for example, [50, 51]) for model loss criteria and computational time. The hidden layers of the ANN are the engine of the algorithm, and the user can only specify the number of hidden layers and the neurones in these layers. In addition to the 'alpha' parameter, the activation function, together with the loss computation methods and epochs are tuned in this study (see Table 1).

2.6 Feature selection and feature transformation

The overtopping datasets with multiple features or dimensions can be challenging for deriving robust ML-based predictions. Highly-dimensional datasets can effectively reduce the efficiency and accuracy of ML algorithms by causing irrelevancy and redundancy in the training process. Redundant data will also significantly increase the computational cost. The problem of data redundancy can be addressed by feature selection methods, aiming to filter a subset of relevant features from a large dataset and largely eliminate redundancy and irrelevancy [52]. The feature selection is basically a permutation combination based on statistical methods to quantify the correlation of each feature with a target feature [53, 54]. For overtopping prediction, den Bieman et al. (2021) adopted feature selection in the form of a permutation analysis of the features.

Feature transformation is another method for extracting meaningful features from a large dataset [55] where the initial number of features is transformed into a new compact dataset while retaining the maximum amount of information possible. Principal Component Analyses is an example of a feature transformation method [56].

This study examines three feature selection methods and one feature transformation method for developing ML models, including Univariate Feature Selection (UFS), Correlation Matrix (CM), Principal Component Analysis (PCA), and Wrapper Methods (WM). In UFS, the most relevant features are ranked based on univariate statistical tests. This method ranks the *k* number of best features according to their random linear dependencies which are measured by the *F-test* score [36]. The CM method measures the correlations between all the features of a dataset with a certain target variable. Similar to UFS, the CM method will rank the best features according to a specific pre-set threshold of the correlation score, and discard all the features with correlation scores below the threshold level. For this study, the threshold correlation value was set at zero to discard any feature that had a negative correlation with the target variable. However, following construction of the correlation map, it was observed that several features in the overtopping dataset were not correlated with other features and the target variable. Hence, the CM was not considered an appropriate method to filter the best features for the wave overtopping dataset used in this study.

PCA is used to determine the variance in large datasets and reduce the dimension of the features in a way to only retain those features that explain the maximum variance in the dataset as a whole. PCA is a method of dimensionality reduction that converts all the correlated variables into principal components that are uncorrelated and explains the maximum variance.

Wrapper methods represent a more complex way of handling feature selection. The methods rely on regression analysis to filter the best subset of features from a large dataset. Due to their complexity, wrapper methods tend to be more time consuming to implement than other feature selection methods [53, 57, 58]. In this study, a wrapper method based on the Ordinary Least Squares technique was applied to the dataset. Following the regression, the *p-values* of the features were considered with a significance level set at 0.05. All features with a *p-value*

exceeding 0.05 were discarded from the overtopping dataset due to the lack of significance in the prediction.

The features that were filtered through PCA and WM were selected for the prediction of wave overtopping from the EurOtop [3] dataset. The results obtained from PCA and WM were in agreement. The PCA identified 20 principal components that could explain the variance in the dataset and the wrapper methods also identified as many as 20 features with a *p-value* less than 0.05. The SVR and ANN models developed in this study rely on the complete datasets (without missing values). For the missing data in the EurOtop dataset, interpolation and k-Nearest Neighbours (kNN) methods were adopted for data imputation. K-Nearest Neighbours (KNN) is a simple machine learning algorithm that can be used for missing value imputation. The basic idea of KNN is to find the K nearest neighbours of an instance with missing values, and to fill in the missing values with the average or median of the values of the nearest neighbours as explained in [59]. The feature selection and imputation methods were applied for the dataset as a pre-processing step and before the data were used as a common input for the ML algorithms.

2.7 Data analysis

The EurOtop database [3] for overtopping at vertical breakwaters, with a total of 1318 data records, was adopted for developing ML-based predictive models. Table 2 describes the dataset parameters and range. The Pearson correlation factor, *r*, of the independent variables with the dependent variable (q) is presented in Table 3. The analysis of the results shows that while no negative correlation in independent variables was observed, the overall degree of correlation between the variables was low. For *r* values of close to zero (at a *p-value* significance level of 0.05), Table 3 indicates no significant correlation between any two variables. The maximum correlation of 0.30 with 'q' was achieved by $T_{m-1,0\ d}$, and the other six independent variables had *r* values ranging from 0.25 to 0.30.

The ML algorithms investigated in this study are capable of handling non-linear relationships between independent and dependent variables. However, low correlations between the variables are indicative of redundant features that need addressing by feature selection and transformation. For the EurOtop dataset [3] used in this study, the entire data was decomposed into 20 principal components, representing the maximum variance in the data that can be explained by 20 features (see Fig 7). Further analysis was carried out to provide insight of the highest ranking features in the PCA. Overall, the analysis shows that 13 out of the 33 features from the dataset can be filtered out without affecting the accuracy of the predictions,

Table 2. Range of overtopping parameters used to extract data for vertical breakwaters.

Overtopping Parameter	Range
$\cot\alpha_d$ (-)	0–0.10
RF	1–3
CF	1–3
h_t (m)	0.05–1.28
B (m)	0
R_c (m)	0.01–1.46
γ_f (-)	1
$H_{m0\ toe}$ (m)	0.03–0.603
q (m^3/s)	$>1.0*10^{-6}$

https://doi.org/10.1371/journal.pone.0289318.t002

Table 3. Correlation table of overtopping parameters.

Pearson *r* Values	Overtopping Parameters
0.3	$T_{m-1,0\ d}$
$0.25 < r < 0.3$	$T_{m\ toe}$, $T_{m\ d}$, $T_{m-1,0t}$, h_t, RF, P_{ow}
$0.20 < r < 0.25$	$H_{m0\ d}$, $T_{p\ d}$, $H_{m0\ toe}$, $T_{p\ toe}$
$r < 0.2$	h_{deep}, mmm, β, h, B_t, $cot\alpha_d$, $cot\alpha_{excl}$, R_c, h_b, A_c, G_c, CF

https://doi.org/10.1371/journal.pone.0289318.t003

while at the same time improving computational efficiency. Following the feature transformation using PCA and feature selection with wrapper methods, the features selected for the analysis include $H_{m0\ d}$, $T_{m\ d}$, $T_{m-1,0\ d}$, mmm, β, h, $H_{m0\ toe}$, $T_{m\ toe}$, $T_{m-1,0t}$, h_t, B_t, $cot\alpha_d$, cot α_u, $cot\alpha_{excl}$, Rc, h_b, Gc, RF, CF and P_{ow} (terms are explained in the glossary). The feature transformation and selection methods trialled different combinations of input data inside their respective algorithms to get the best subset of the features. Hence, it was not required to perform any further trials for identifying the best combination of input parameters. The features extracted from feature transformation and selection are directly fed into the analysis.

A train-test data split of 70%-30% was adopted in the development of the ML models in this study, with 70% of the data being used to train the models and the remaining 30% of the data used to predict the dependent variable, '*q*' in the testing phase. Following completion of the prediction process, the predicted values of overtopping discharges were compared to the measured values reported in the EurOtop dataset [3] which were kept hidden from the algorithms during the testing phase. The analysis of the ML results was mainly carried out using Scikit-learn library [36]. The flowchart of the methodology framework is described in Fig 8.

Following train-test split, the models were optimized by hyperparameter tuning, feature transformation and selection, cross-validation, and Grid Search. The model outputs include

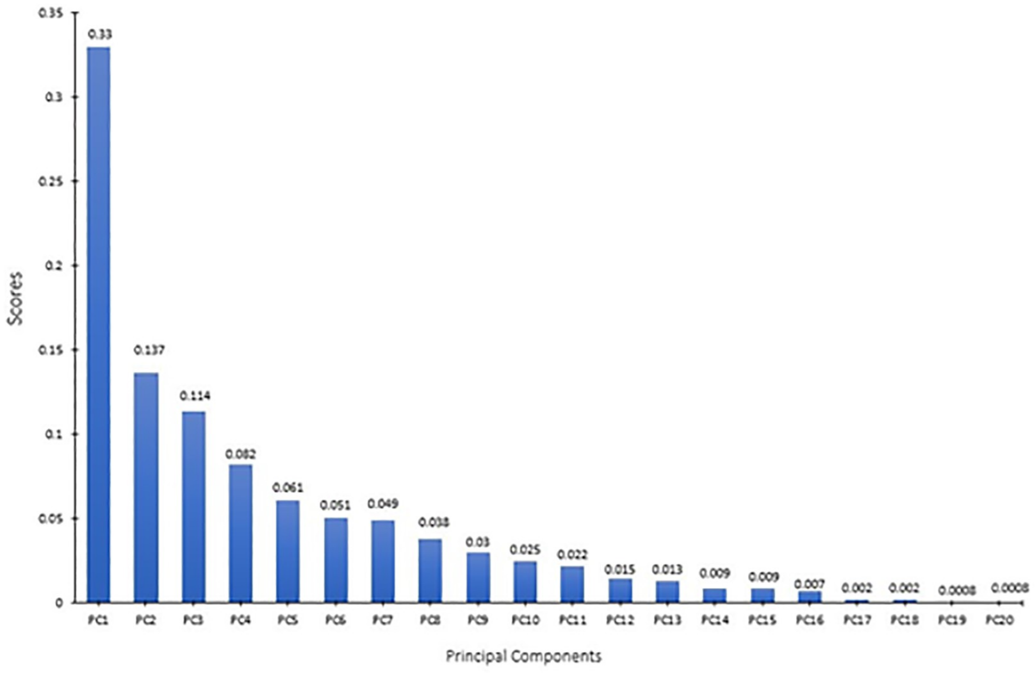

Fig 7. PCA Decomposition and scores of the dataset.

https://doi.org/10.1371/journal.pone.0289318.g007

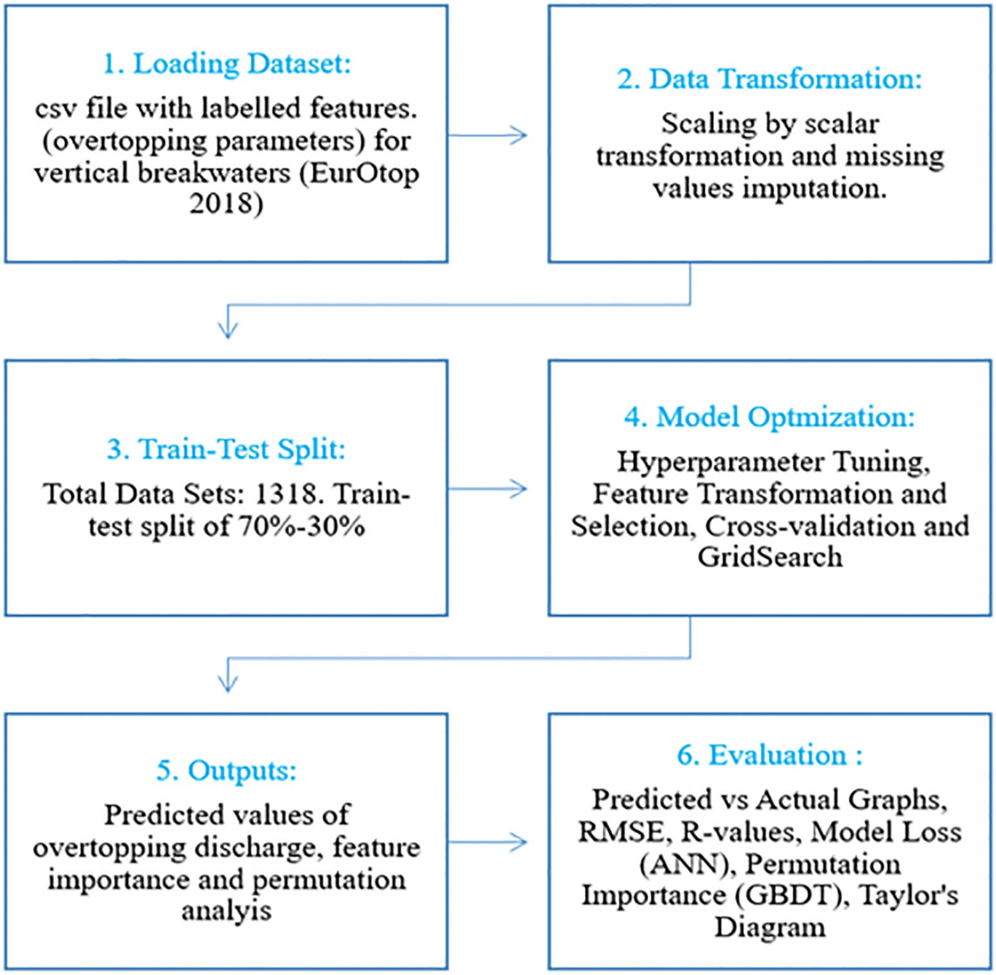

Fig 8. Framework of the methodology.

https://doi.org/10.1371/journal.pone.0289318.g008

the predicted overtopping rates, together with ancillary information such as feature importance and permutation analysis (described in Section 3). A detailed statistical analysis was then performed to evaluate the performance of the proposed ML-based overtopping prediction models for vertical breakwaters.

2.8 Empirical estimation of overtopping

The mean overtopping rates obtained from the algorithms were compared with corresponding rates estimated from empirical calculations.

According to [3], the mean overtopping rate (q) for non-impulsive conditions at vertical walls is estimated by Eq 13:

$$\frac{q}{\sqrt{gH_{m_0}}} = 0.05exp\left(-2.78\frac{R_c}{H_{m_0}}\right) \quad (13)$$

where, H_{m_0} is the significant wave height from spectral analysis, R_c is the crest freeboard and g is the gravitational acceleration. The non-impulsive and impulsive conditions are deduced by

Eqs 14 and 15, respectively, as suggested by [60].

$$\frac{h_t^2}{(H_{m_0}L_{m-1,0})} > 0.23 \quad (14)$$

$$\frac{h_t^2}{(H_{m_0}L_{m-1,0})} \leq 0.23 \quad (15)$$

where h_t denotes the water depth at the toe of the structure and $L_{m-1,0}$ is the wave-length of the waves in deep water calculated from $\frac{gT_{m-1,0}^2}{2\pi}$ with $T_{m-1,0}$ referring to the spectral wave period. The mean overtopping rate for impulsive wave conditions is calculated from Eqs 16 and 17, according to the given conditions [60].

$$\frac{q}{\sqrt{gH_{m_0}}} = 0.011\left(\frac{H_{m_0}}{h_t s_{m-1,0}}\right)^{0.5} exp\left(-2.2\frac{R_c}{H_{m_0}}\right), \text{ for } 0 < \frac{R_c}{H_{m_0}} < 1.35 \quad (16)$$

$$\frac{q}{\sqrt{gH_{m_0}}} = 0.0014\left(\frac{H_{m_0}}{h_t s_{m-1,0}}\right)^{0.5}, for \frac{R_c}{H_{m_0}} \geq 1.35 \quad (17)$$

where $S_{m-1,0}$ is the wave steepness, calculated from $\frac{H_{m_0}}{L_{m-1,0}}$.

For the case of 'no influencing foreshore' in front of the structure, Eq 18 as proposed by EurOtop [3], is employed.

$$\frac{q}{\sqrt{gH_{m_0}}} = 0.047 \exp\left[-\left(2.35\frac{R_c}{H_{m_0}}\right)^{1.3}\right] \quad (18)$$

3. Results and discussion

3.1 Prediction results

Following extraction and filtration of overtopping data for the case of vertical seawalls from the EurOtop database [3], hyperparameter tuning, feature transformation and selection were conducted, and the selected overtopping features were inputted to the proposed ML algorithms (i.e. Random Forest, Gradient Boosted Decision Trees, Support Vector Machines Regression, and Artificial Neural Network). The dimensionless predicted values of 'q' $\left(\frac{q\_predicted}{\sqrt{9.81*(H_{mtoe})^3}}\right)$ were compared against the dimensionless measured values of 'q' $\left(\frac{q\_measured}{\sqrt{9.81*(H_{mtoe})^3}}\right)$ to evaluate the predictive performance of the proposed ML models for wave overtopping. The overall results of statistical analysis indicate that all the ML algorithms examined in this study are capable of predicting 'q' with high accuracy. Predicted overtopping values are compared to those measured in Fig 9. It is to note that empirical equations (Eq 13, Eqs 16–18) are based on best fitting to laboratory and field measurements at vertical walls, and this study compares the performance of four developed ML models against the measured values as reported in EurOtop database (i.e., laboratory and field measurements of overtopping rates at vertical walls).

Analysis of the results indicates that for the four ML algorithms tested, the majority of predictions for small overtopping volumes are within the 95% confidence interval, although the

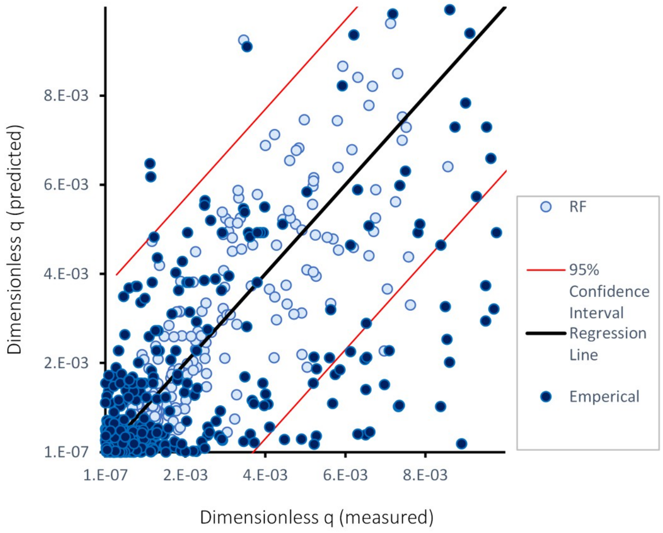

Fig 9. Comparison of the dimensionless q (predicted) $\left(=\frac{q\_predicted}{\sqrt{9.81*(H_{mtoe})^3}}\right)$ and dimensionless q (measured) $\left(=\frac{q\_measured}{\sqrt{9.81*(H_{mtoe})^3}}\right)$ for—a) RF, b) ANN, c) GBDT, and d) SVR.

https://doi.org/10.1371/journal.pone.0289318.g009

accuracy of the predictions reduces for larger overtopping rates. The predictions from the SVR models are shown to exhibit slightly more scatter from the regression line compared to those for the RF and GBDT models, while predictions from the ANN show greater scatter. Data therefore suggests that ANN is the least accurate predictor algorithm amongst the four models tested in this study. The performance of ML models is further explained by the statistical scores in Table 4.

The results obtained for the RF and GBDT models show better agreement to measured overtopping rates, as evidenced by the number of predicted data points within the 95% confidence interval, compared to the predictions from the ANN and SVR models (see Fig 9). The mean overtopping rates calculated from empirical equations, described in Section 2.8 and adapted from EurOtop [3], yielded results that exhibited more scatter when plotted against the measured overtopping rates. Comparable scatter was also evident in the study of [34]. This observation suggests that the mean overtopping rates obtained from empirical equations are less accurate than the rates deduced from the algorithms. All the algorithms examined here have yielded strong correlation scores. As for computational efficiency, DT based algorithms

Table 4. Evaluation scores of ML algorithms for five key statistical metrices.

Algorithm	Coefficient of Determination- R^2	Root Mean Square Error- RMSE	Root Mean Square Logarithmic Error- RMSLE	Mean Absolute Error (MAE)	Relative Absolute Error (RAE)
RF	0.84	0.0025	0.00247	0.0010	0.27
GBDT	0.81	0.0027	0.0026	0.0011	0.27
SVR	0.80	0.0027	0.0027	0.0011	0.28
ANN	0.75	0.0031	0.0032	0.0014	0.36

https://doi.org/10.1371/journal.pone.0289318.t004

such as RF and GBDT outperformed SVR and ANN. Under the given hyperparameter conditions (see Table 1), the prediction task took 27, 29, 48 and 502 seconds to complete for GBDT, RF, SVR and ANN respectively, for a computer with a Central Processing Unit of 8 cores, 16 gigabytes of Random Access Memory and 6 gigabytes of dedicated video memory. This indicates that RF, GBDT and SVR are performing the prediction task more efficiently than ANN, and also with improved accuracy.

3.2 Evaluation metrics

The wave overtopping prediction results obtained from all the four ML algorithms were evaluated and compared using 5 statistical metrics for non-dimensional values of mean overtopping rate, q. (see Table 4). The results show that the best prediction performance was achieved by the RF model with an R^2 value of 0.84. The RMSE and RMSLE values were 0.0025 and 0.00247, respectively, indicating the cumulative number of outliers in the RF model. The RMSE measure is taken to be the most effective parameter for analysing the performance of ML model predictions (following [61]). The MAE and RAE measures for RF model were 0.0010 and 0.27, respectively. GBDT was found to produce the second-best predictions for wave overtopping. A coefficient of determination (R^2) of 0.81 indicates that the GBDT model exhibited similar performance to the RF algorithm. The RMSE and RMSLE for GBDT models were slightly higher than for the RF model, with values of 0.0027 and 0.0026, respectively. The MAE and RAE determined for GBDT predictions were 0.0011 and 0.27, respectively, which are close to those values measured for the RF model.

The feed-forward and back-propagation ANN produced good predictions but achieved a 0.75 R^2 score which is lower than other ML models tested in this study. The lower R^2 score for the ANN model is confirmed by higher RMSE and RMSLE values of 0.0031 and 0.0032, respectively. The MAE and RAE scores are also higher than for the other algorithms at 0.0014 and 0.36, respectively.

In terms of the statistical metrics, it can be inferred that the RF, GBDT and SVR models performed better than the ANN model in prediction of wave overtopping from the EurOtop database. For the RF, GBDT, and SVR models, the R^2 scores are above 0.8 while for the ANN model, this is 0.75. The RMSE for ANN is higher than for the other models, which suggests that the standard deviation of the residuals for ANN is larger than the other three algorithms. RAE reflects the measure of the relative error in predicted values and in this score, ANN shows the highest error of 0.36 or 36%, indicating more error in the predictions from the ANN model compared to the RF, GBDT and SVR models.

3.3 Feature importance and physical consistency

Feature importance analysis was conducted following the prediction of overtopping discharge '*q*', to compare the consistencies of the tested ML algorithms in the prediction tasks. Feature importance shows the features (i.e., parameters) that are most influential in the prediction tasks. Absence or presence of these features will significantly alter the overtopping prediction accuracy. For the same dataset, different ML algorithms should show similar feature importance to cross-validate the consistency of the prediction results. The GBDT and RF algorithms perform feature importance analysis by default, while the SVR and ANN algorithms are not readily capable of this process. Hence, permutation importance analysis was performed for SVR and ANN using ELI5 [62]. However, both the feature importance analysis (for GBDT and RF) and ELI5 (for SVR and ANN) indicate similar results, a quantitative measure of the influence of the features for a robust prediction performance. Feature importance values are expressed in the range of 0 to 1 in order of increasing significance for the prediction tasks. The

permutation importance is independently scored for each feature, which is randomly taken from the prediction tasks and the resulting change in accuracy is measured on a range of 0 to 1 in order of increasing significance of accuracy. For all the ML algorithms, $H_{\mathrm{m0\ toe}}$ and R_c were the top two important features in the prediction tasks. Features such as cotα_{excl} and $H_{\mathrm{m0\ d}}$ are also amongst the top 5 important features in multiple algorithms. These results suggest that although the prediction tasks are data driven, and all the algorithms performed independently, there is some agreement between the features that were found to be the most influential in these algorithms. This confirms the consistency of data analytics that form the core of the algorithms examined in this study. The finding that significant wave height at the toe of the structure and the crest freeboard of the structure have been identified as the most important parameters in the prediction of overtopping discharge by ML algorithms is consistent with observations from physical modelling. The feature importance analysis results for the RF and GBDT, and the permutation importance analysis results for the SVR and ANN algorithms are shown in Fig 10.

Although some new features are highlighted as influential in the permutation importance analysis of ANN and SVR (Fig 10), the top two features are similar to the results obtained for the RF and GBDT models, indicating consistency in the prediction tasks.

In addition, further investigation was carried out to gain knowledge on the consistency of the predicted overtopping quantities with the physics of the overtopping process. For this, the two most important features, the crest freeboard and height of wave at the toe of the structure are considered. The variations of dimensionless overtopping rates to the ratio of the crest freeboard and wave height at the toe of the structure ($R_c/H_{m0,t}$) is decreasing (Fig 11), indicating that the predictive results align with our understanding of physical processes, and increasing $R_c/H_{m0,t}$ reduces the overtopping rates.

Recent studies [11, 63] and [12] (65, 12, have suggested that shallow foreshore slope can have a considerable impact on wave behaviour and subsequently wave overtopping, although previous studies have typically focused only on the effects of deep-water wave characteristics on this process. This is exemplified in the work of [64] where it was found that deep-water parameter-based wave overtopping models were in good agreement with field measurements of wave overtopping at a studied site in the United Kingdom. Therefore, foreshore slope was expected to be more influential in the feature importance analysis in this work. However, from

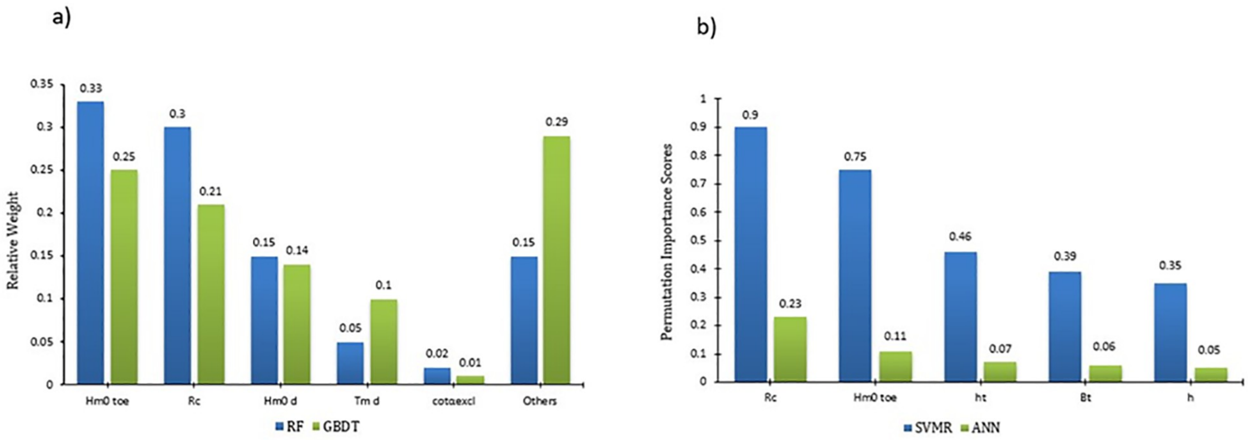

Fig 10. a) Feature importance of the RF and GBDT and b) Permutation Importance analysis of SVR and ANN.

https://doi.org/10.1371/journal.pone.0289318.g010

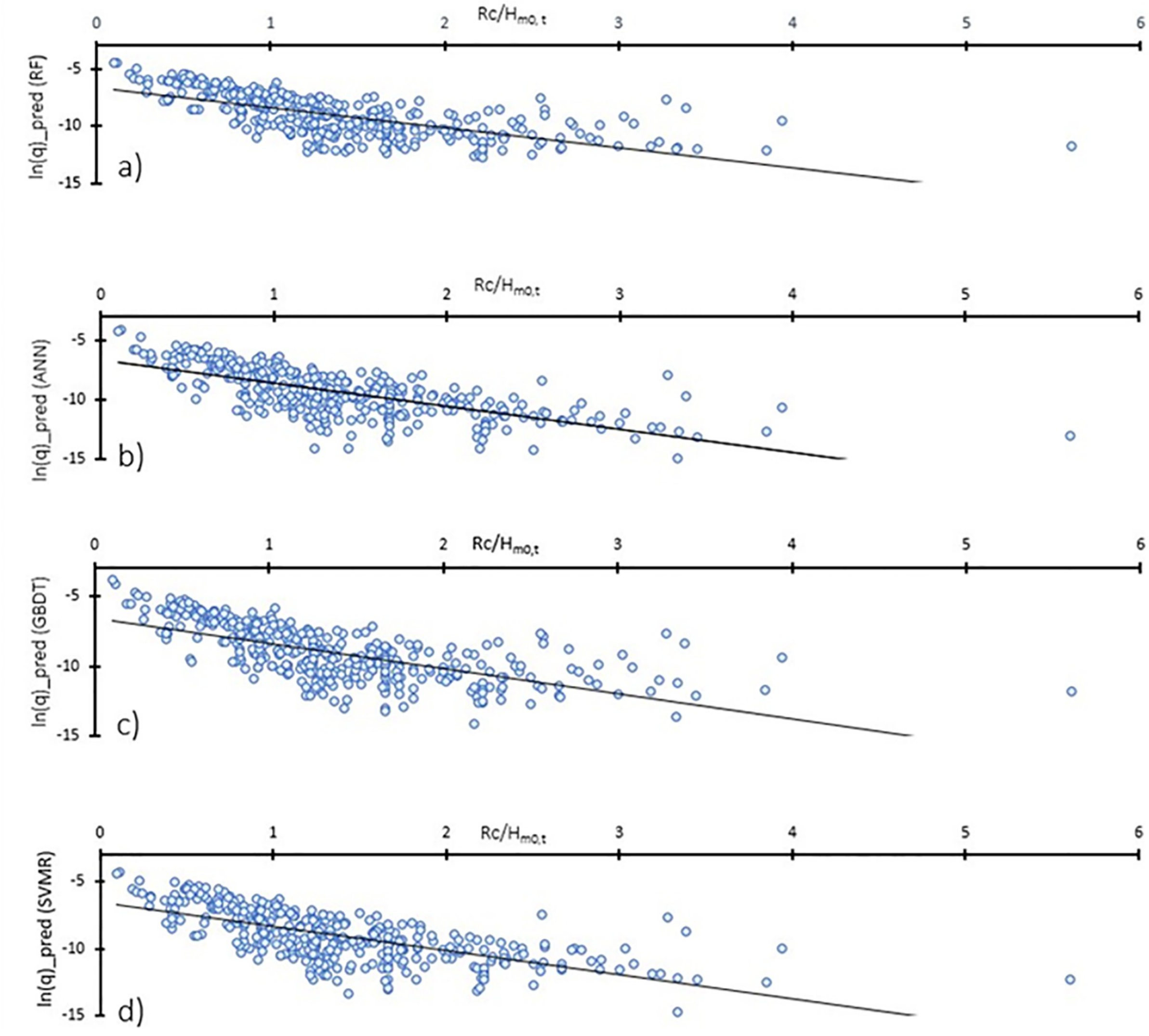

Fig 11. Variation of dimensionless overtopping rate 'q' with Rc/$H_{m0,t}$ for a) RF, b) ANN, c) GBDT, and d) SVR.

https://doi.org/10.1371/journal.pone.0289318.g011

Fig 10, this was shown not to be the case. The resulting findings of feature importance (foreshore slope) are in-line with results for permutation importance previously reported by [27, 28] for overtopping prediction at sloping structures where the authors did not report any considerable impacts of foreshore slope in overtopping predictions. This might be linked to the fact that the advanced ML models developed and employed in this study for overtopping predictions are capable of automatically understanding the complex and co-dependency of parameters by assigning weights during the training phase. Nevertheless, it is to be noted that feature importance is not a guarantee or a sole representative of improved model accuracy, and of the influence of feature selection on model accuracy can depend on many factors, including the size and complexity of the data, the type of model being used, and the relationships between the features and the target variable (see, [65] and [59]). The evidence presented thus far suggests that while feature importance can be useful in evaluating the relevance of features, it should be used in conjunction with other evaluation metrics, such as cross-validation

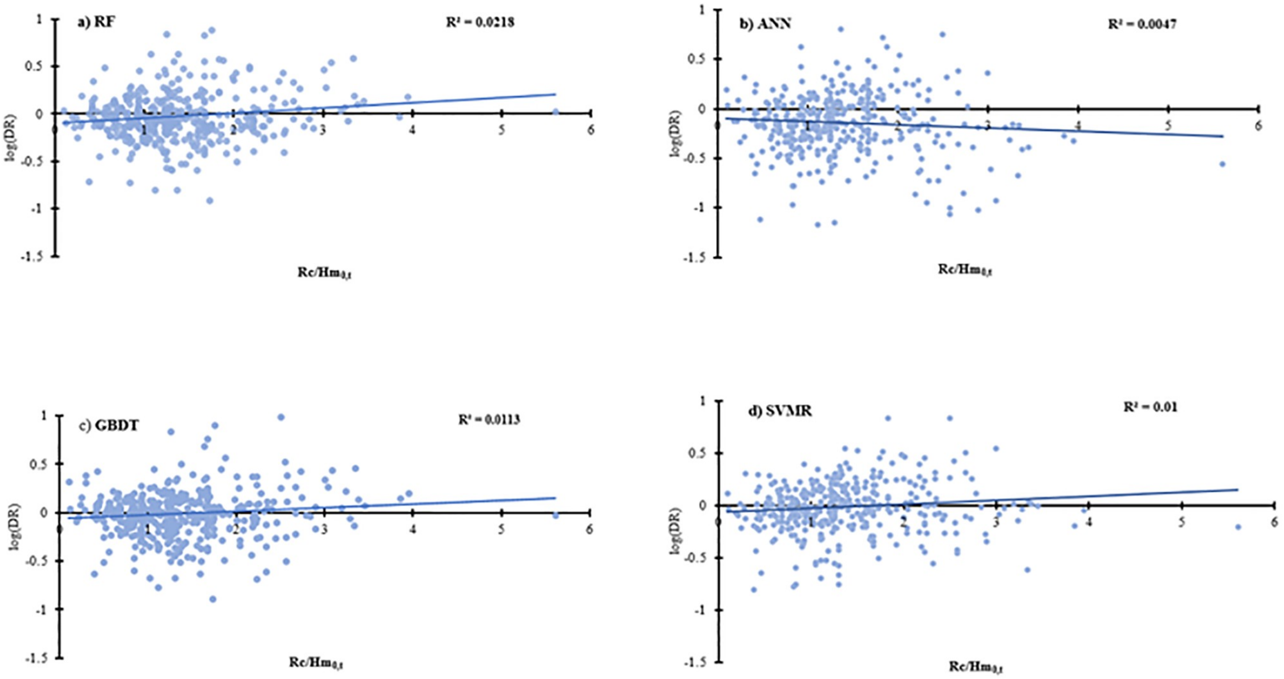

Fig 12. Variation of discrepancy ratio (DR) with $R_c/H_{m0,t}$ for a) RF, b) ANN, c) GBDT, and d) SVR.

https://doi.org/10.1371/journal.pone.0289318.g012

and statistical scores as adopted in this work, to ensure that the model is robust and generalizes well to unseen data.

The discrepancy ratio (DR) between the actual and the predicted values is plotted against $R_c/H_{m0,t}$ in Fig 12. To ensure robust predictions, the DR should be independent of the input data [66]. For all the algorithms, the DR results are shown to be independent from the two most important features in the dataset with very low R^2 values. Hence, it can be concluded that the results from the ML algorithms can be well interpreted in the physical context of overtopping and the results are also robust with regards to the discrepancy ratio.

3.4 Taylor's diagram

The Taylor's Diagram is an efficient and graphical approach to represent the statistical metrics from prediction results [34]. Three statistical parameters including the correlation coefficient plotted as azimuthal angle (in black), centered Root Mean Square (cRMS) values plotted radially (in green), and the standard deviations plotted horizontally (in blue) are represented in the Taylor' Diagram (Fig 13).

Analysis of Fig 13 shows that the RF model has the best predictive performance for wave overtopping amongst the four ML algorithms that were examined in this study. RF results have the lowest standard deviation and cRMS, and the highest correlation. The GBDT and SVR have similar performance in terms of the standard deviation, with the former exhibiting a slightly greater cRMS and lower correlation coefficient. The models are better separated in terms of correlation coefficient compared to the standard deviation. The ANN, although having similar correlation coefficients compared to the GBDT and SVR algorithms, exhibits increased standard deviation. This means the residual errors in ANN are greater than in the other models examined. Overall, therefore, ANN is ranked after the GBDT and SVR

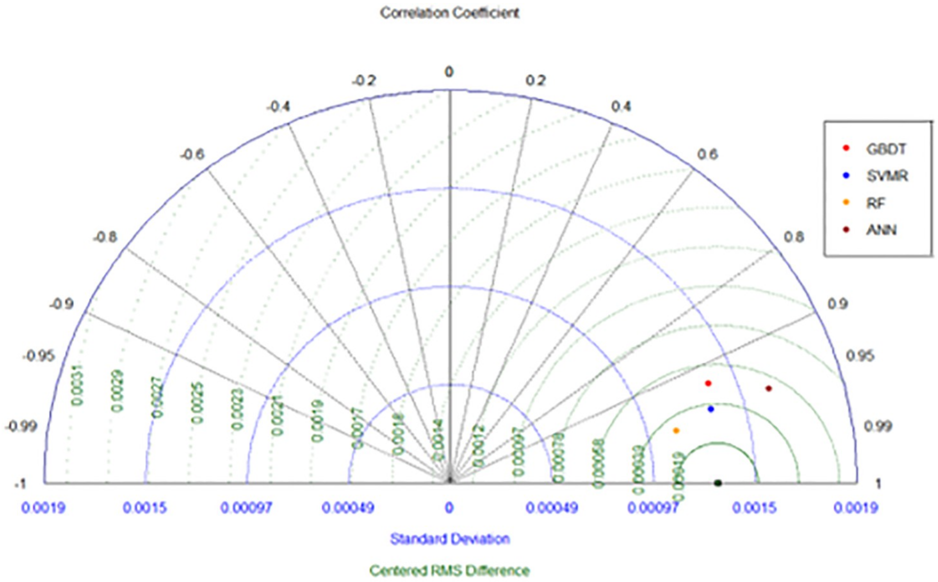

Fig 13. Taylor's diagram of the statistical scores from the prediction analysis of the four ML algorithms.

https://doi.org/10.1371/journal.pone.0289318.g013

algorithms. On the other hand, the RF model has the lowest standard deviation coupled with the highest correlation score. Considering the statistical scores, the RF outperforms other ML models tested here, while ANN generates relatively less accurate results in comparison to the others. The results from Taylor's diagram highlight that the RF model is the most accurate ML model amongst the models tested here, for the prediction of wave overtopping at vertical breakwaters.

ML algorithms are characterised by different advantages and disadvantages and a generalized recommendation on which ML technique can provide a superior performance is difficult to opine. The performance comparison of ML algorithms depends on the individual applications and the structure of data [67]. For the case of wave overtopping prediction from vertical breakwaters based on the EurOtop database [3], the findings of this study suggest that decision tree-based models (i.e., GBDT and RF) perform better than ANNs. This is consistent with the findings of [68] and [69], where [68] draws attention to the capabilities of DT models to map the non-linear relationships between the input and target features. [67] found that following hyperparameter tuning and feature selection, SVR performed better than ANNs. This can be attributed to the ability of SVR to better tackle the noise in the input data [70]. Amongst the DT-based models tested here, RF slightly outperforms the GBDT. The performance of MLs vary according to datasets and for cases where the input dataset has missing values, high dimensionality, and is collective in nature (such as [3]); the RF technique was shown to perform better than GBDT, which is in line with the findings of existing studies (e.g. [71]). The feature importance analysis scores of RF are also shown to be moderately more accurate than those of GBDT [72, 73].

In this study, Machine learning algorithms were shown to offer improved accuracy over empirical methods for predicting varve overtopping at vertical seawalls. ML algorithms can learn patterns and relationships in large amounts of data, which can then be used to make more accurate predictions of overtopping. Hyperparameter tuning enables the models to curtail to specific datasets. This is especially useful when the underlying physical processes are complex and difficult to model using traditional methods. Although, the underlying physics is

not explicitly described in the ML models, the feature importance analysis can deduce the most important physical parameters in overtopping as demonstrated in this study. The models in this study therefore, present a holistic approach that combines feature selection, hyperparameter tuning and feature selection to predict overtopping.

4. Conclusions

This study compared the appropriateness and robustness of advanced machine learning techniques for prediction of wave overtopping at vertical seawalls. Four ML algorithms including RF, GBDT, SVR, and ANN were developed for prediction of overtopping discharges. The developed ML models were applied to a large and comprehensive dataset of overtopping from EurOtop 2018. A methodological approach to pre-processing that includes steps for missing value imputations, feature transformation (PCA) and selection (wrapper methods), and data scaling, was adopted, prior to application of ML-based predictive modelling of wave overtopping. These steps ensured that performance of the algorithms was not affected by missing values and redundancy of data. The training process was performed by hyperparameter tuning so that the individual algorithms were fine-tuned or curtailed according to the specific dataset features. Cross validation was conducted for the training step so that the ML algorithms could trial on a random subset of training data before being applied on the test set. The train-test split of 70%-30% was used following standard ML modelling protocols. These measures ensured that a uniform framework was followed to achieve comparable results from the predictions performed by the four algorithms. Feature importance and permutation importance analysis identified water depth at the toe of the structure and crest freeboard as the two most important features for overtopping prediction, confirming the relevancy of the results to the physical processes that govern overtopping. Parametric analysis was performed to identify the relationship of dimensionless overtopping quantity '*q*' with the ratio of the crest freeboard and the wave height at the toe of the structure, $R_c/H_{m0,t}$. The decreasing trend of the overtopping quantity with $R_c/H_{m0,t}$ highlights that ML algorithms are capable of understanding the physical overtopping process.

Comprehensive statistical error indexes including R^2, RMSE, RMSLE, MAE, and RAE were determined for all the four tested ML algorithms. The RF algorithm outperformed other tested algorithms across all the statistical scores with a R^2 value of 0.84, RMSE of 0.0025, RMSLE of 0.00247 and MAE, RAE values of 0.0010 and 0.27, respectively. The GBDT and SVR models yielded R^2 values of 0.81 and 0.80, respectively, which are close to that of RF, but the RMSE was 8% higher for SVR and GBDT, respectively. The higher residual errors in SVR and GBDT were also evident in the Taylor's diagram that was prepared, where the two algorithms had higher standard deviations than RF. The ANN model exhibited the least accurate predictions of wave overtopping with a lower R^2 value and higher residual errors. The DT based models performed significantly better than ANN in terms of computational time. This was reflected by the time taken for GBDT and RF to complete the prediction tasks in 27 and 29 seconds, respectively. The time taken for SVR to complete the prediction task was 48 seconds. This indicates that although ANN is a more widely used and endorsed ML method to predict overtopping quantities, other ML algorithms, especially DT-based models and SVNR, can perform better. One common advantages of RF, GBDT, and SVR, in contrast to ANNs used in this study, is the capability of more fine-tuning provisions based on the overtopping dataset provisions for vertical breakwaters. Therefore, further research should follow to investigate the applicability of RF, GBDT and SVR for overtopping predictions from defences with different geometrical configurations and data sources. Overall, the application of ML algorithms in overtopping predictions from coastal infrastructures is still limited. Although the ML

algorithms tested here differed in terms of evaluation metrices and statistical scores, their feature importance/permutation analysis yielded consistent results. Also, all the algorithms examined in this study understood the underlying physical processes governing the wave overtopping from vertical breakwaters. The overtopping rates from the algorithms were more accurate than those derived from empirical methods.

The methodological framework proposed for comparing the appropriateness and robustness of ML algorithms for wave overtopping prediction enables a fair comparison of ML models. The trade-offs of the models are discussed in terms of statistical metrics. Feature transformation methods (i.e., PCA) and advanced feature selection methods (i.e., wrapper method) are, for the first time, implemented in this study to enhance the overtopping prediction from ML-based methods. The use of feature selection methods has significantly reduced the time-consuming trials of selecting the best set of features, without the loss of important information. The proposed hyperparameter tuning for RF and SVR algorithms provides additional enhancement to the existing ML-based studies for wave overtopping predictions. This study, for the first time, successfully implements a RF algorithm for predicting wave overtopping from plain vertical seawalls based on the EurOtop database [3]. The comparative study shows that RF consistently outperform all other three ML algorithms tested here.

Like other ML-based predictive studies for wave overtopping, the presence of missing values in the dataset and validity of the ML model for the range of training parameters are the main limitations of this study.

Acknowledgments

We sincerely thank Professor Jentsje W. Van der Meer, Professor Barbara Zanuttigh and Dr Sara Mizar Formentin for providing the EurOtop overtopping database [3].

Author Contributions

Conceptualization: M. A. Habib, J. J. O'Sullivan, S. Abolfathi, M. Salauddin.

Data curation: M. A. Habib.

Formal analysis: M. A. Habib.

Funding acquisition: M. Salauddin.

Investigation: M. A. Habib.

Methodology: M. A. Habib, M. Salauddin.

Project administration: M. Salauddin.

Resources: M. Salauddin.

Software: M. A. Habib.

Supervision: J. J. O'Sullivan, S. Abolfathi, M. Salauddin.

Validation: M. A. Habib.

Visualization: M. A. Habib.

Writing – original draft: M. A. Habib.

Writing – review & editing: M. A. Habib, J. J. O'Sullivan, S. Abolfathi, M. Salauddin.

References

1. Temmerman S, Meire P, Bouma TJ, Herman PMJ, Ysebaert T, De Vriend HJ. Ecosystem-based coastal defence in the face of global change. Nature. 2013 Dec 5; 504(7478). https://doi.org/10.1038/nature12859 PMID: 24305151
2. Intergovernmental Panel on Climate Change. Climate Change 2021 –The Physical Science Basis [Internet]. Cambridge University Press; 2023. https://www.cambridge.org/core/product/identifier/9781009157896/type/book
3. Van der Meer, J.W., Allsop, N.W.H., Bruce, T., De Rouck, J., Kortenhaus, A., Pullen, T., et al. Manual on wave overtopping of sea defences and related structures. An overtopping manual largely based on European research, but for worldwide application [Internet]. 2018. www.overtopping-manual.com
4. Victor L, van der Meer JW, Troch P. Probability distribution of individual wave overtopping volumes for smooth impermeable steep slopes with low crest freeboards. Coast Eng. 2012 Jun; 64.
5. O'Sullivan J, Salauddin M, Abolfathi S, Pearson J. Effectiveness of eco-retrofits in reducing wave overtopping on seawalls. Coast Eng Proc [Internet]. 2020 Dec 28;(36v):13. Available from: https://journals.tdl.org/icce/index.php/icce/article/view/10170
6. Salauddin M, O'Sullivan JJ, Abolfathi S, Peng Z, Dong S, Pearson JM. New insights in the probability distributions of wave-by-wave overtopping volumes at vertical breakwaters. Scientific Reports. 2022 Sep 28; 12(1):16228. https://doi.org/10.1038/s41598-022-20464-5 PMID: 36171253
7. Salauddin M, O'Sullivan JJ, Abolfathi S, Pearson JM. Eco-Engineering of Seawalls—An Opportunity for Enhanced Climate Resilience From Increased Topographic Complexity.Front Mar Sci [Internet]. 2021 Jun 16; 8. Available from: https://www.frontiersin.org/articles/10.3389/fmars.2021.674630/full
8. Dong S, Salauddin M, Abolfathi S, Tan ZH, Pearson JM. The influence of geometrical shape changes on wave overtopping: a laboratory and sph numerical study. In: Coasts, Marine Structures and Breakwaters 2017. ICE Publishing; 2018. https://doi.org/10.1680/cmsb.63174.1217
9. Dang B-L, Nguyen-Xuan H, Abdel Wahab M. Numerical study on wave forces and overtopping over various seawall structures using advanced SPH-based method. Eng Struct. 2021 Jan; 226.
10. Ravindar R, Sriram V, Salauddin M. Numerical modelling of breaking wave impact loads on a vertical seawall retrofitted with different geometrical configurations of recurve parapets. J Water Clim Chang [Internet]. 2022 Oct 1; 13(10):3644–74. Available from: https://iwaponline.com/jwcc/article/13/10/3644/90986/Numerical-modelling-of-breaking-wave-impact-loads
11. Lashley CH, Bricker JD, van der Meer J, Altomare C, Suzuki T. Relative magnitude of infragravity waves at coastal dikes with shallow foreshores: A prediction tool. J Waterw Port, Coastal, Ocean Eng [Internet]. 2020 Sep; 146(5). Available from: https://ascelibrary.org/doi/10.1061/%28ASCE%29WW.1943-5460.0000576
12. Yuhi M, Mase H, Kim S, Umeda S, Altomare C. Refinement of integrated formula of wave overtopping and runup modeling. Ocean Eng [Internet]. 2021 Jan; 220:108350. Available from: https://linkinghub.elsevier.com/retrieve/pii/S0029801820312592
13. Franco L, de Gerloni M, van der Meer JW. Wave Overtopping on Vertical and Composite Breakwaters. In: Coastal Engineering 1994. New York, NY: American Society of Civil Engineers; 1995.
14. Dong S, Abolfathi S, Salauddin M, Pearson JM. Spatial distribution of wave-by-wave overtopping behind vertical seawall with recurve retrofitting. Ocean Engineering. 2021 Oct 15; 238:109674.
15. Salauddin M, Pearson J. A laboratory study on wave overtopping at vertical seawalls with a shingle foreshore. Coast Eng Proc [Internet]. 2018 Dec 30;(36):56. Available from: https://icce-ojs-tamu.tdl.org/icce/index.php/icce/article/view/8330
16. Chau K. A review on integration of artificial intelligence into water quality modelling. Mar Pollut Bull [Internet]. 2006 Jul; 52(7):726–33. Available from: https://linkinghub.elsevier.com/retrieve/pii/S0025326X06001366 https://doi.org/10.1016/j.marpolbul.2006.04.003 PMID: 16764895
17. Gong B, Im J, Jensen JR, Coleman M, Rhee J, Nelson E. Characterization of Forest Crops with a Range of Nutrient and Water Treatments Using AISA Hyperspectral Imagery. GIScience Remote Sens [Internet]. 2012 Jul 15; 49(4):463–91. Available from: https://www.tandfonline.com/doi/full/10.2747/1548-1603.49.4.463
18. Kumar L, Afzal MS, Afzal MM. Mapping shoreline change using machine learning: a case study from the eastern Indian coast. Acta Geophys [Internet]. 2020 Aug 30; 68(4):1127–43. Available from: https://link.springer.com/10.1007/s11600-020-00454-9
19. Rhee J, Im J, Carbone GJ, Jensen JR. Delineation of climate regions using in-situ and remotely-sensed data for the Carolinas. Remote Sens Environ [Internet]. 2008 Jun; 112(6):3099–111. Available from: https://linkinghub.elsevier.com/retrieve/pii/S0034425708000862.

20. Altomare C, Laucelli DB, Mase H, Gironella X. Determination of Semi-Empirical Models for Mean Wave Overtopping Using an Evolutionary Polynomial Paradigm. J Mar Sci Eng [Internet]. 2020 Jul 29; 8 (8):570. Available from: https://www.mdpi.com/2077-1312/8/8/570

21. Abbas YB, Hossein E, Naser S, Bahareh K, Abolfathi S. Machine Learning as a Downscaling Approach for Prediction of Wind Characteristics under Future Climate Change Scenarios. Complexity. 2022; 2022:13. Available from: https://doi.org/10.1155/2022/8451812

22. Donnelly J, Abolfathi S, Pearson J, Chatrabgoun O, Daneshkhah A. Gaussian process emulation of spatio-temporal outputs of a 2D inland flood model. Water Res [Internet]. 2022 Oct; 225:119100. Available from: https://linkinghub.elsevier.com/retrieve/pii/S0043135422010466 https://doi.org/10.1016/j.watres.2022.119100 PMID: 36155010

23. Noori R, Ghiasi B, Salehi S, Esmaeili Bidhendi M, Raeisi A, Partani S, et al. An Efficient Data Driven-Based Model for Prediction of the Total Sediment Load in Rivers. Hydrology [Internet]. 2022 Feb 17; 9 (2):36. Available from: https://www.mdpi.com/2306-5338/9/2/36

24. Ghiasi B, Noori R, Sheikhian H, Zeynolabedin A, Sun Y, Jun C, et al. Uncertainty quantification of granular computing-neural network model for prediction of pollutant longitudinal dispersion coefficient in aquatic streams. Sci Rep [Internet]. 2022 Mar 17; 12(1):4610. Available from: https://doi.org/10.1038/s41598-022-08417-4 PMID: 35301353

25. Van Gent MRA, van den Boogaard HFP, Pozueta B, Medina JR. Neural network modelling of wave overtopping at coastal structures. Coast Eng. 2007 Aug; 54(8).

26. Verhaeghe H, De Rouck J, van der Meer J. Combined classifier–quantifier model: A 2-phases neural model for prediction of wave overtopping at coastal structures. Coast Eng. 2008 May; 55(5).

27. den Bieman JP, Wilms JM, van den Boogaard HFP, van Gent MRA. Prediction of Mean Wave Overtopping Discharge Using Gradient Boosting Decision Trees. Water. 2020 Jun 15; 12(6). Available from: https://doi.org/10.3390/w12061703

28. den Bieman JP, van Gent MRA, van den Boogaard HFP. Wave overtopping predictions using an advanced machine learning technique. Coast Eng. 2021 Jun; 166. Available from: https://doi.org/10.1016/j.coastaleng.2020.103830

29. De Rouck J, Geeraerts J, Troch P, Kortenhaus A, Pullen T, Franco L. CLASH Final Report, Full Scientific and Technical Report, Ghent University, Belgium. [Internet]. 2005. http://resolver.tudelft.nl/uuid:80d718d2-b89d-435b-8741-3f1c582ea4bb

30. Kambekar AR, Deo MC. Wave simulation and forecasting using wind time history and data-driven methods. Ships Offshore Struct [Internet]. 2010 Sep 2; 5(3):253–66. Available from: http://www.tandfonline.com/doi/abs/10.1080/17445300903439223

31. Etemad-Shahidi A, Mahjoobi J. Comparison between M5′ model tree and neural networks for prediction of significant wave height in Lake Superior. Ocean Eng. 2009 Nov; 36(15–16). Available from: https://doi.org/10.1016/j.oceaneng.2009.08.008

32. Jafari E, Etemad-Shahidi A. Derivation of a New Model for Prediction of Wave Overtopping at Rubble Mound Structures. J Waterw Port, Coastal, Ocean Eng. 2012 Jan; 138(1). Available from: https://doi.org/10.1061/(ASCE)WW.1943-5460.0000099

33. Chen S, Chen H, Peng C, Wang Y, Hu Y. Analysis of Factors Influencing Wave Overtopping Discharge from Breakwater Based on an MIV-BP Estimation Model. Water [Internet]. 2022 Sep 21; 14(19):2967. Available from: https://www.mdpi.com/2073-4441/14/19/2967

34. Hosseinzadeh S, Etemad-Shahidi A, Koosheh A. Prediction of mean wave overtopping at simple sloped breakwaters using kernel-based methods. J Hydroinformatics [Internet]. 2021 Sep 1; 23(5):1030–49. Available from: https://iwaponline.com/jh/article/23/5/1030/83455/Prediction-of-mean-wave-overtopping-at-simple

35. Habib MA, O'Sullivan JJ, Salauddin M. Prediction of wave overtopping characteristics at coastal flood defences using machine learning algorithms: A systematic rreview. IOP Conf Ser Earth Environ Sci [Internet]. 2022 Sep 1; 1072(1):012003. Available from: https://iopscience.iop.org/article/10.1088/1755-1315/1072/1/012003

36. Pedregosa F, Varoquaux G, Gramfort A, Michel V, Thirion B, Grisel O, et al. Scikit-learn: Machine Learning in Python. 2012 Jan 2; Available from: http://arxiv.org/abs/1201.0490

37. Kotu V, Deshpande B. Classification. In: Predictive Analytics and Data Mining [Internet]. Elsevier; 2015. p. 63–163. https://linkinghub.elsevier.com/retrieve/pii/B9780128014608000045

38. Sutton CD. Classification and Regression Trees, Bagging, and Boosting. In 2005. p. 303–29. https://linkinghub.elsevier.com/retrieve/pii/S0169716104240111

39. Elbeltagi A, Pande CB, Kouadri S, Islam ARMT. Applications of various data-driven models for the prediction of groundwater quality index in the Akot basin, Maharashtra, India. Environ Sci Pollut Res [Internet]. 2022 Mar 20; 29(12):17591–605. Available from: https://link.springer.com/10.1007/s11356-021-17064-7

40. Elbeltagi A, Kumari N, Dharpure J, Mokhtar A, Alsafadi K, Kumar M, et al. Prediction of Combined Terrestrial Evapotranspiration Index (CTEI) over Large River Basin Based on Machine Learning Approaches. Water [Internet]. 2021 Feb 20; 13(4):547. Available from: https://www.mdpi.com/2073-4441/13/4/547

41. Awad M, Khanna R. Efficient Learning Machines [Internet]. Berkeley, CA: A press; 2015. http://link.springer.com/10.1007/978-1-4302-5990-9

42. Roushangar K, Koosheh A. Evaluation of GA-SVR method for modeling bed load transport in gravel-bed rivers. J Hydrol [Internet]. 2015 Aug; 527:1142–52. Available from: https://linkinghub.elsevier.com/retrieve/pii/S0022169415004199

43. McCulloch WS, Pitts W. A logical calculus of the ideas immanent in nervous activity. Bull Math Biophys [Internet]. 1943 Dec; 5(4):115–33. Available from: http://link.springer.com/10.1007/BF02478259

44. Zanuttigh B, Formentin SM, van der Meer JW. Prediction of extreme and tolerable wave overtopping discharges through an advanced neural network. Ocean Eng [Internet]. 2016 Nov; 127:7–22. Available from: https://linkinghub.elsevier.com/retrieve/pii/S0029801816304127

45. EurOtop. Wave overtopping of sea defences and related structures: assessment manual. 2007. www.overtopping-manual.com.

46. Formentin SM, Zanuttigh B, van der Meer JW. A Neural Network Tool for Predicting Wave Reflection, Overtopping and Transmission. Coast Eng J. 2017 Mar 10; 59(1). Available from: https://doi.org/10.1142/S0578563417500061

47. Babaee M, Maroufpoor S, Jalali M, Zarei M, Elbeltagi A. Artificial intelligence approach to estimating rice yield*. Irrig Drain [Internet]. 2021 Oct 14; 70(4):732–42. Available from: https://onlinelibrary.wiley.com/doi/10.1002/ird.2566

48. Cheng C-L, Shalabh, Garg G. Coefficient of determination for multiple measurement error models. J Multivar Anal [Internet]. 2014 Apr; 126:137–52. Available from: https://linkinghub.elsevier.com/retrieve/pii/S0047259X14000141

49. Huang D-S, Yu H-J. Normalized Feature Vectors: A Novel Alignment-Free Sequence Comparison Method Based on the Numbers of Adjacent Amino Acids. IEEE/ACM Trans Comput Biol Bioinforma [Internet]. 2013 Mar; 10(2):457–67. Available from: http://ieeexplore.ieee.org/document/6466328/ https://doi.org/10.1109/TCBB.2013.10 PMID: 23929869

50. LeCun Y, Bengio Y, Hinton G. Deep learning. Nature [Internet]. 2015 May 28; 521(7553):436–44. Available from: https://www.nature.com/articles/nature14539 https://doi.org/10.1038/nature14539 PMID: 26017442

51. Xavier G, Yoshua B. Understanding the difficulty of training deep feedforward neural networks. In: Proceedings of the Thirteenth International Conference on Artificial Intelligence and Statistics. 2010. https://proceedings.mlr.press/v9/glorot10a.html

52. Cai J, Luo J, Wang S, Yang S. Feature selection in machine learning: A new perspective. Neurocomputing [Internet]. 2018 Jul; 300:70–9. Available from: https://linkinghub.elsevier.com/retrieve/pii/S0925231218302911

53. Blum AL, Langley P. Selection of relevant features and examples in machine learning. Artif Intell [Internet]. 1997 Dec; 97(1–2):245–71. Available from: https://linkinghub.elsevier.com/retrieve/pii/S0004370297000635

54. Liu H. Motoda H. Feature Selection for Knowledge Discovery and Data Mining, Springer Science & Business Media, 2012.

55. Liu H, Motoda H. Feature Transformation and Dimensionality Reduction. In: Feature Selection for Knowledge Discovery and Data Mining [Internet]. Boston, MA: Springer US; 1998. p. 151–87. http://link.springer.com/10.1007/978-1-4615-5689-3_6

56. Roessner U, Nahid A, Chapman B, Hunter A, Bellgard M. Metabolomics—The Combination of Analytical Biochemistry, Biology, and Informatics. In: Comprehensive Biotechnology [Internet]. Elsevier; 2011. p. 435–47. https://linkinghub.elsevier.com/retrieve/pii/B9780444640468000276

57. Vergara JR, Estévez PA. A review of feature selection methods based on mutual information. Neural Comput Appl [Internet]. 2014 Jan 13; 24(1):175–86. Available from: http://link.springer.com/10.1007/s00521-013-1368-0

58. Kohavi R, John GH. Wrappers for feature subset selection. Artif Intell [Internet]. 1997 Dec; 97(1–2):273–324. Available from: https://linkinghub.elsevier.com/retrieve/pii/S000437029700043X

59. Hastie T, Tibshirani R, & Friedman J. The elements of statistical learning: data mining, inference, and prediction (Vol. 2). 2009. Springer

60. Van der Meer J, Bruce T. New Physical Insights and Design Formulas on Wave Overtopping at Sloping and Vertical Structures. J Waterw Port, Coastal, Ocean Eng [Internet]. 2014 Nov; 140(6). Available from: https://ascelibrary.org/doi/10.1061/%28ASCE%29WW.1943-5460.0000221

61. Yaseen Z, Ehteram M, Sharafati A, Shahid S, Al-Ansari N, El-Shafie A. The Integration of Nature-Inspired Algorithms with Least Square Support Vector Regression Models: Application to Modelling River Dissolved Oxygen Concentration. Water [Internet]. 2018 Aug 23; 10(9):1124. Available from: http://www.mdpi.com/2073-4441/10/9/1124.

62. Korobov M. Welcome to ELI5's documentation! 2017. Welcome to ELI5's documentation!—ELI5 0.11.0 documentation

63. Hofland B, Chen X, Altomare C, Oosterlo P. Prediction formula for the spectral wave period T m-1,0 on mildly sloping shallow foreshores. Coast Eng [Internet]. 2017 May; 123:21–8. Available from: https://linkinghub.elsevier.com/retrieve/pii/S037838391630237X

64. Lashley CH, Brown JM, Yelland MJ, van der Meer JW, Pullen T. Comparison of deep-water-parameter-based wave overtopping with wirewall field measurements and social media reports at Crosby (UK). Coast Eng [Internet]. 2023 Jan; 179:104241. Available from: https://linkinghub.elsevier.com/retrieve/pii/S0378383922001545

65. James G, Witten D, Hastie T, & Tibshirani R. An introduction to statistical learning. 2013 (Vol. 112). Springer.

66. Sahay RR, Dutta S. Prediction of longitudinal dispersion coefficients in natural rivers using genetic algorithm. Hydrol Res [Internet]. 2009 Dec 1; 40(6):544–52. Available from: https://iwaponline.com/hr/article/40/6/544/961/Prediction-of-longitudinal-dispersion-coefficients

67. Bisgin H, Bera T, Ding H, Semey HG, Wu L, Liu Z, et al. Comparing SVM and ANN based Machine Learning Methods for Species Identification of Food Contaminating Beetles. Sci Rep [Internet]. 2018 Dec 25; 8(1):6532. Available from: http://www.nature.com/articles/s41598-018-24926-7 https://doi.org/10.1038/s41598-018-24926-7 PMID: 29695741

68. Jun M-J. A comparison of a gradient boosting decision tree, random forests, and artificial neural networks to model urban land use changes: the case of the Seoul metropolitan area. Int J Geogr Inf Sci [Internet]. 2021 Nov 2; 35(11):2149–67. Available from: https://www.tandfonline.com/doi/full/10.1080/13658816.2021.1887490

69. Du G, Shin KJ, Yuan L, Managi S. A comparative approach to modelling multiple urban land use changes using tree-based methods and cellular automata: the case of Greater Tokyo Area. Int J Geogr Inf Sci [Internet]. 2018 Apr 3; 32(4):757–82. Available from: https://www.tandfonline.com/doi/full/10.1080/13658816.2017.1410550

70. Burges CJCA. Tutorial on Support Vector Machines for Pattern Recognition. Data Mining and Knowledge Discovery. 1998. 2, 121–167.

71. Yang F, Wang H, Mi H, Lin C, Cai W. Using random forest for reliable classification and cost-sensitive learning for medical diagnosis. BMC Bioinformatics [Internet]. 2009 Jan 30; 10(S1):S22. Available from: https://bmcbioinformatics.biomedcentral.com/articles/10.1186/1471-2105-10-S1-S22 PMID: 19208122

72. Zhao T, Liu S, Xu J, He H, Wang D, Horton R, et al. Comparative analysis of seven machine learning algorithms and five empirical models to estimate soil thermal conductivity. Agric For Meteorol [Internet]. 2022 Aug; 323:109080. Available from: https://linkinghub.elsevier.com/retrieve/pii/S0168192322002684

73. Salauddin M, Shaffrey D, Habib MA. Data-driven approaches in predicting scour depths at a vertical seawall on a permeable shingle foreshore. Journal of Coastal Conservation. 2023 Jun; 27(3):18.
